# Attribution of recent temperature behaviour reassessed by a neural-network method

**DOI:** 10.1038/s41598-017-18011-8

**Published:** 2017-12-15

**Authors:** Antonello Pasini, Paolo Racca, Stefano Amendola, Giorgio Cartocci, Claudio Cassardo

**Affiliations:** 1 Institute of Atmospheric Pollution Research, National Research Council, Rome, Italy; 2 Department of Economics and Statistics, University of Turin, Torino, Italy; 3 Department of Mathematics and Physics, Roma Tre University, Rome, Italy; 4 Department of Physics, University of Turin, Torino, Italy; 5 Department of Atmospheric Science and Engineering, Ewha Womans University, Seoul, Korea

## Abstract

Attribution studies on recent global warming by Global Climate Model (GCM) ensembles converge in showing the fundamental role of anthropogenic forcings as primary drivers of temperature in the last half century. However, despite their differences, all these models pertain to the same dynamical approach and come from a common ancestor, so that their very similar results in attribution studies are not surprising and cannot be considered as a clear proof of robustness of the results themselves. Thus, here we adopt a completely different, non-dynamical, data-driven and fully nonlinear approach to the attribution problem. By means of neural network (NN) modelling, and analysing the last 160 years, we perform attribution experiments and find that the strong increase in global temperature of the last half century may be attributed basically to anthropogenic forcings (with details on their specific contributions), while the Sun considerably influences the period 1910–1975. Furthermore, the role of sulphate aerosols and Atlantic Multidecadal Oscillation for better catching interannual to decadal temperature variability is clarified. Sensitivity analyses to forcing changes are also performed. The NN outcomes both corroborate our previous knowledge from GCMs and give new insight into the relative contributions of external forcings and internal variability to climate.

## Introduction

GCMs are the standard tools for catching the complexity of climate system and simulating its behaviour^1,2^. In particular, in the framework of this virtual laboratory we are able to perform attribution experiments (e.g., by setting some forcings to their preindustrial values) in order to understand which factors have mainly influenced the behaviour of some variable of climatic importance, such as the global temperature, in the recent past. The results of these experiments (through ensemble runs of different GCMs) clearly indicate that anthropogenic forcings (primarily greenhouse gas forcing) are the main responsible for the recent global warming^3^.

May we consider the convergence of attribution results from these models as a proof of robustness of the results themselves? This is a critical point, due to the fact that the political choices to contrast climate change are based on the claim that anthropogenic forcings are the fundamental causes of the recent global warming. This problem has been recently analysed and discussed^4,5^. In short, to achieve robustness we need to obtain a common result from independent means of investigation (models) and GCMs do not seem so independent from each other, because they are all historically linked together by a common origin and relations of mutual generation, and follow the same methodological dynamical scheme.

Thus, attribution results from different approaches could be compared with GCMs’ ones for understanding if we have robust results. Previous studies using data-driven models, such as neural networks and Granger causality analyses (see bibliography in ref.^5^), suggest a dominant role of anthropogenic forcings, but a comprehensive comparison (following the same rationale used in GCMs’ experiments) is lacking.

In this framework, basing on the quite recent application of NNs in atmospheric and climate sciences^6,7^, a NN model, specifically developed for modelling relationships among variables in small datasets^8^ and previously applied in a simplified form to global/regional attribution studies^9–11^, is adopted here for an attribution analysis of the recent global warming.

We focus on annual data since the middle of the 19^th^ century related to global temperature T (HadCRUT4 time series) and radiative forcings (RF) of: greenhouse gases (RFGHG), black carbon (RFBC), anthropogenic sulphates (RFSOX), solar activity (RFSOLAR), and volcanoes (RFVOL): see Supplementary Fig. S1. Here, RFANTH (=RFGHG+RFBC+RFSOX) is considered the anthropogenic forcing, while RFSOLAR and RFVOL represent the natural forcings. Besides, also data about indices of natural variability (see Supplementary Fig. S2) are taken into account: Southern Oscillation Index (SOI) – related to El Niño Southern Oscillation (ENSO) –, Pacific Decadal Oscillation (PDO) and Atlantic Multidecadal Oscillation (AMO). See Methods for more details.

Feedforward NNs^12,13^ are simple tools for performing multiple nonlinear regressions and assessing the influences of external forcings – considered as inputs of the networks – on the global temperature (the target to be “approached” by the networks’ output). In doing so, however, one has to avoid overfitting problems and handle the sources of variability (e.g., the random choices for initial weights and validation sets). This leads to consider for our investigation a tool^8^ that maximises the information for training in a context of quite short records available (about 160 years), minimises overfitting problems and averages away the NN model variability by multiple runs and an ensemble approach. See Methods for the specific features of this model and more details on its “ensemble” application.

## Reconstruction of T and attribution via neural network modelling

Generally, previous studies about NN attribution^9,14,15^ aimed at understanding how much variance of the global temperature record could be explained by anthropogenic or natural forcings separately, through the analysis of the reconstruction performance of NN models endowed with one of the two different kinds of forcing as input. However, due to the powerful capacity of NNs to fit the data, this does not assure reliable results and it is not possible to compare these outcomes with the attribution ones by GCMs.

Thus, we follow the rationale of dynamical modelling, where one chooses some validated models and simulates the behaviour of global T by supposing that certain forcings remain fixed at their preindustrial levels. Analogously, now we search for some NN models that are able to well reconstruct the temperature time series once considered all observed values of forcings as inputs, then apply their transfer functions (the validated NN models) to new inputs, in which we mimic the fact that some of their values show no trend since 1850, finally obtaining new outputs in terms of “simulated” T. In this case one can appreciate the roles of the real changes in different forcings on the behaviour of T.

**Figure 1:**
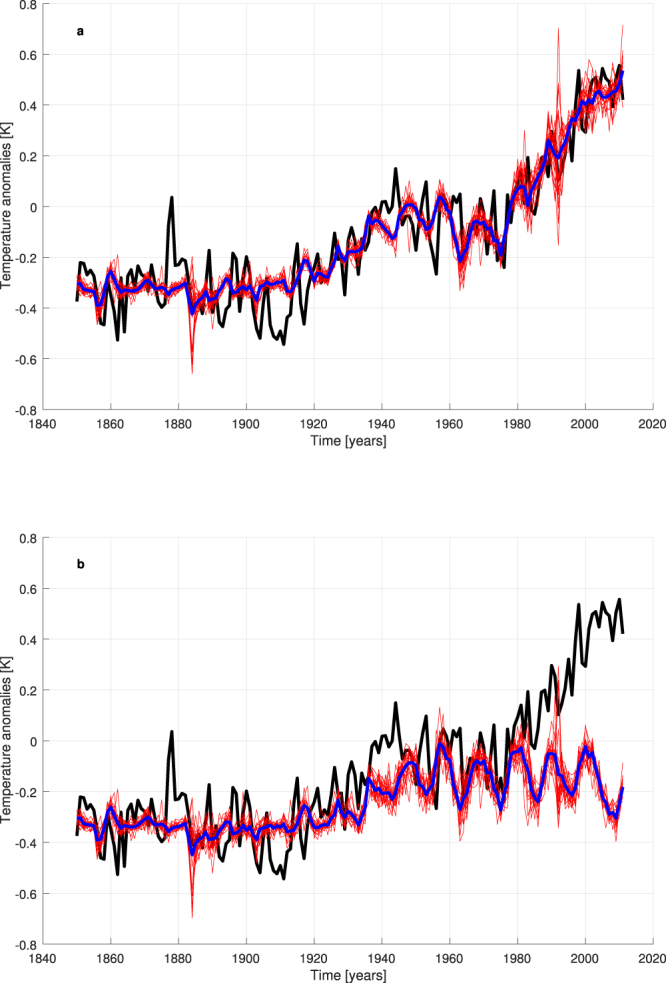
Reconstruction of global T by NN models. Black line = observed T, red lines = results of ensemble runs, blue line = ensemble mean. (**a**) With real values of RFANTH, RFSOLAR and RFVOL as inputs. (**b**) Attribution runs when RFANTH is fixed at its value of 1850.

Each model ensemble consists of 20 NNs that supply temperature reconstructions for each year considered as the test set in a generalized leave-one-out training-validation-test procedure (see Methods). These networks are endowed with 3 different inputs (the complete set of natural and anthropogenic forcings: RFANTH, RFSOLAR, RFVOL), 4 hidden neurons and one output (to be compared with the annual global T, i.e. the target): the number of hidden neurons has been empirically fixed and keep the networks small enough to avoid overfitting. Figure 1a shows the good performance of the NN models in reconstructing the global T (R = 0.913 and RMSE = 0.109 K for the ensemble mean). In particular, the ensemble mean well follows the trends of the various periods. Just few data are underestimated or overestimated, such as the relative maxima around 1878 and 1944, and the minimum around 1910. Interannual and decadal variability are partially caught, too. A high variability for ensemble runs is visible in 1884 and 1992, the years following the two most intense volcanic episodes, but this is understandable if one looks at the “spot-like” time series of RFVOL, because the reconstructions of these two years generally require big extrapolations from RFVOL values presented to the NNs in the training set.

Thus, we consider that these NN models are able to catch the nonlinear mixing of influences of the various forcings on T and supply us with an ensemble of validated models, so that the attribution activity may start. Note that, even if we mainly deal with the reconstruction performance of the ensemble mean, that tends to hide nonlinear influences, the NN results presented in this study are always better than those coming from multilinear regressions using the same data, so that nonlinearities play however a role.

### Specific attribution results

The first attribution experiment is the standard one: what happens if the anthropogenic forcing is set to preindustrial levels? Here we start by taking the validated NN models (networks endowed with fixed connection weights), for each year we put the observed inputs of natural forcings related to that year and the values of RFANTH fixed at 1850 (the first year of our dataset), and finally we propagate the forcing signal from inputs to output through the NNs. The result is presented in Fig. 1b, where it is evident that the recent increase in temperature disappears, being replaced by a constant temperature trend since 1960. This picture shows a fine similarity with the well-known Fig. 10.1(b) of ref.^3^, in which the GCMs’ approach to attribution is adopted. Now, with a completely different approach, essentially the same result is obtained: anthropogenic forcings had a fundamental role in causing the recent global warming.

Of course, the various anthropogenic forcings have different magnitudes in terms of RFs, also as influence signs on warming (e.g., sulphates have a cooling effect). Thus, it would be interesting to investigate their separated roles in determining the temperature curve. One could substitute the single RFANTH input with the three anthropogenic RFs (RFGHG, RFBC and RFSOX) as distinct inputs, and repeat the previous attribution exercise for each forcing separately. In doing so, however, we should build larger NNs and this could lead to overfitting problems, due to the short time series available^16^. Thus, a distinct approach is followed.

**Figure 2:**
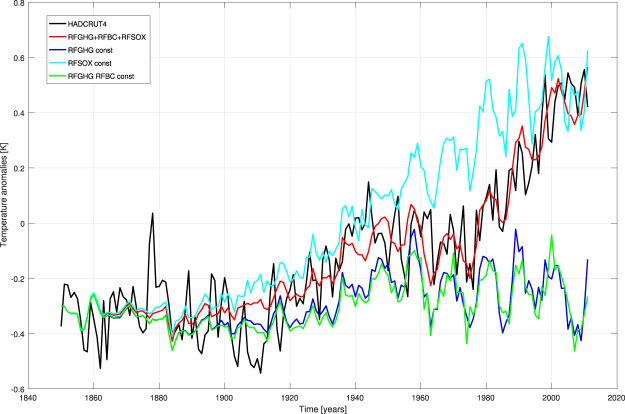
Warming and cooling roles of the distinct anthropogenic forcings by a NN attribution experiment. Black line = observed T, red line = reconstructed T by all inputs with actual values, blue, light blue and green lines = attribution runs with constant forcing(s).

We consider the difference between the ensemble-mean time series of reconstructed T in the full reconstruction runs (Fig. 1a) and in the attribution experiment when RFANTH is kept constant at its value of 1850 (Fig. 1b). Then, we reconstruct this residual time series (which approximates the influence of the total anthropogenic RF on T) by NNs endowed with the three anthropogenic forcings as inputs, and an attribution exercise can be performed as above, by separately keeping each forcing constant at its 1850 value (see Supplementary Fig. S3). Once the results of this attribution experiment are added to the temperature time series reconstructed in Fig. 1b, we can explore the specific roles of warming and cooling factors in different periods (see Fig. 2).

Thus, we can appreciate in more details the great influence of GHGs (and, at a lesser extent, BC) on the temperature of the last half century. Note also that, in this period, the curves obtained supposing GHGs (and BC) constant are lower than the “attribution curve” in Fig. 1b by about 0.1 K, because here a cooling factor (RFSOX) has been considered with its real pattern. Furthermore, without changes in RFSOX, the hiatus during the period 1945–1975 would not have appeared and the increasing trend in temperature would have been monotonic. The maximum influence of RFGHG on T is about 0.9 K during the last years of the period considered here, while the highest perturbation to T by RFSOX is about 0.3 K at the end of the cited hiatus.

In this framework, what is the importance of natural forcings? Certainly, the solar radiation (with its 11-year period) influenced the decadal variability. However, the time series of RFSOLAR also shows the transition from a low-power regime to a high-power one during the period 1910–1950 (see Supplementary Fig. S1b). Has this phenomenon influenced the temperature time series during the recent warming or in other periods? This is also investigable (for the first time, at our knowledge) by our NN models. In particular, a synthetic time series of RFSOLAR (which is “stationary” and does not show that transition) has been built in terms of a first-order Fourier series based on the first 65 years available (see RFSOLSTAT in Supplementary Fig. S1b).

**Figure 3:**
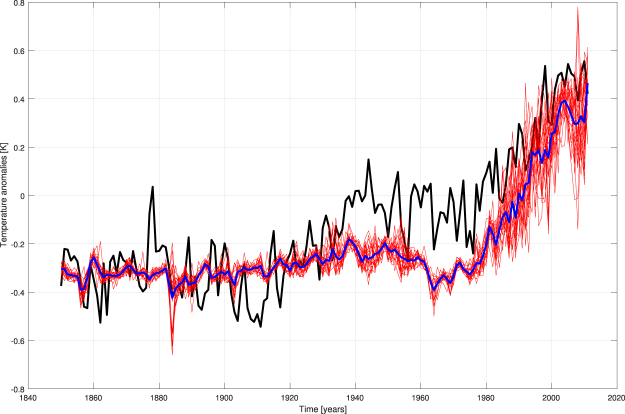
Attribution experiment for solar influence. Black line = observed T, red lines = results of ensemble runs, blue line = ensemble mean. We mimic a stationary solar forcing by putting RFSOLSTAT in input to validated NN models.

Thus, once considered the validated NN models, the influence of Sun on T can be studied by putting the new yearly values of RFSOLSTAT in input, transferring them through the fixed connection weights and looking at the new outputs. The results are sketched in Fig. 3. Here, the increasing trend in T during the years 1910–1945 is not caught with a stationary power from Sun and during the 1945–1975 hiatus T is about 0.2 K lower than observed. The “stationary Sun” has no detectable influence on the value of the increasing T trend of the last period.

In general, the influence of a forcing factor on the temperature behaviour in a certain period is bigger when its attribution curve in Figs 2 and 3 is more distant from the real temperature curve. From a more quantitative point of view, on all the period of investigation, the calculation of Root Mean Square Error (RMSE) can help (see Supplementary Table S1).

### Analysing the role of natural variability

**Figure 4:**
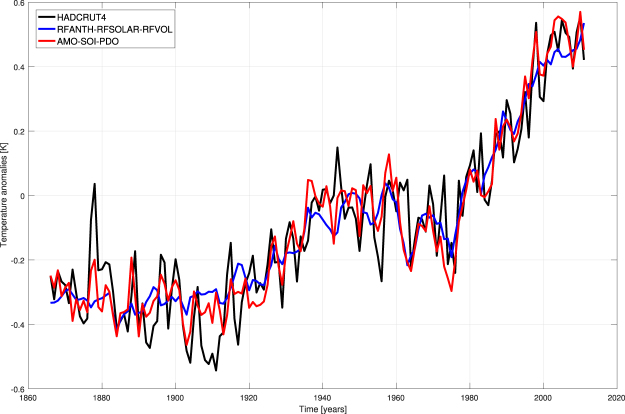
Reconstruction of the global temperature using also data about circulation patterns. Black line = observed T, blue line = ensemble mean from NN models with external forcings as inputs, red line = ensemble mean when the performance of the NN models with indices of natural variability as inputs is added to the previous result. Note that the graph starts in 1866 because data about SOI are not available before that year.

Thus, in this NN framework we are able to explain about 83% of the variance (R^2^) of the global T curve through external forcings (NNs’ inputs). Can the residual variance be explained, at least in part, by natural variability? To explore this topic, we took the time series of the residuals of “observed T minus reconstructed T” (ensemble mean) in Fig. 1a and tried to reconstruct this time series by NNs endowed with three indices of natural variability as inputs: AMO, SOI and PDO. The results show that our NNs reconstruct the residuals in a satisfying manner (see Supplementary Fig. S4a). Once the ensemble mean reconstruction of residuals is added to the ensemble mean reconstruction of the real T curve obtained through external forcings, this latter curve is better “simulated”: R = 0.944, RMSE = 0.089 K, so that the explained variance is now about 89%. In particular (see Fig. 4), the better performance in catching interannual and decadal variability of the temperature curve is evident along all the period of investigation, and in the 1945–1975 hiatus period, in particular. This also leads to mitigate the overemphasized role of the 11-year solar cycle on temperature variability in previous NN models fed with just external forcings.

A further investigation can be performed on the roles of the single circulation patterns. The attribution strategy can be adopted once more by considering the validated NN models on the residuals and by feeding them in input with real values for two indices and a constant value for the remaining third index. In doing so, one discovers the big influence of AMO in catching both interannual and decadal variability of the temperature signal. This is consistent with other results in literature^17–19^, attesting its role of major driver of natural variability on the planet. ENSO appears to contribute especially to interannual variability (when it is taken constant, decadal variability is however caught), while PDO seems to not contribute substantially to the signal of global T (see Supplementary Fig. S4b–d).

Obviously, our approach disregards the possible influences and relationships among the changes in the external forcings and the behaviour of these circulation patterns. In our framework, this could be considered assuming forcings and indices as inputs of the same NNs, so putting them at the same level.

One could insert the three indices as inputs together with external forcings. In doing so, however, we should build sensibly larger NNs and this could lead to overfitting problems. Thus, due to the great influence of AMO on T, just discovered, we choose to furtherly investigate its role, by adding only AMO as input in our NN models, together with external forcings.

**Figure 5:**
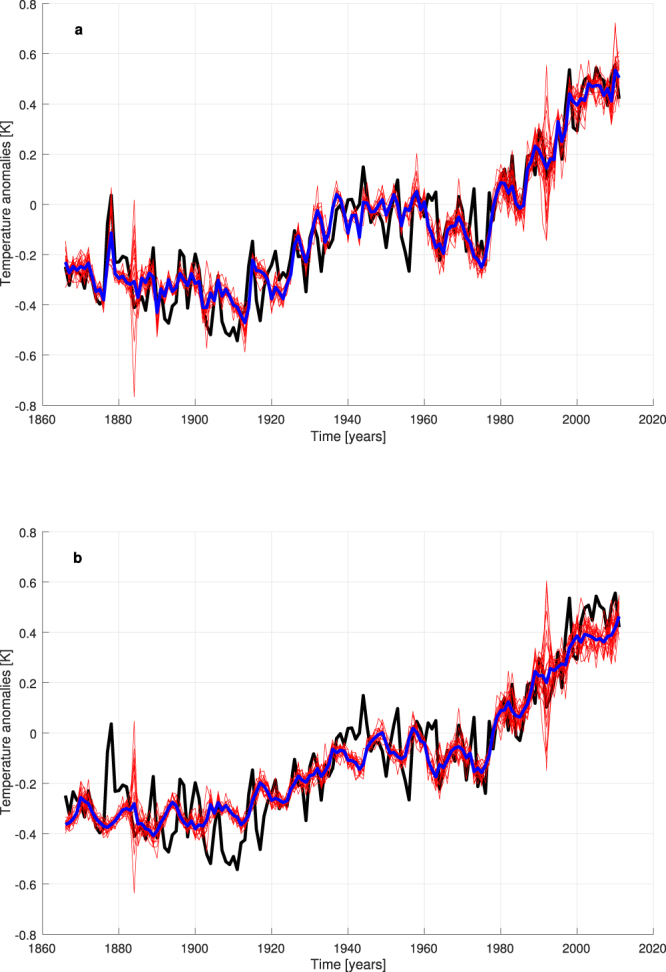
Reconstruction of global T by NN models with AMO as input. Black line = observed T, red lines = results of ensemble runs, blue line = ensemble mean. (**a**) With real values of RFANTH, RFSOLAR, RFVOL and AMO as inputs. (**b**) Attribution runs when AMO is kept fixed at its mean value.

The reconstruction performance of the ensemble mean obviously benefits from the direct inclusion of AMO as input, substantially showing the same performance (R = 0.943 and RMSE = 0.089 K) reached by the previous “residual strategy” when all the three indices were considered. However, the most notable result is obtained from the attribution proof performed by keeping AMO constant at its mean value. In this case, multi-decadal variability has been caught less well: this is evident especially in the T increasing period 1910–1945 and in the following hiatus period 1945–1975 (see Fig. 5). In particular, this shows that probably AMO had a role in the cited hiatus period, a role that we can superimpose to that coming from variations in sulphates. We will analyse better this problem in the next Sections.

## Sensitivity analyses

### Uncertainties in forcing factors

In this paper, data from reliable sources are used as forcing factors. However, notable uncertainties are still present in these data, especially for those forcings that act in both direct and indirect manner on climate, such as sulphates and black carbon. Thus, some sensitivity studies are performed to analyse if our main results about attribution are robust, that is still valid even when we explore a variability in these forcings that can represent our range of uncertainties.

To do so, the results of NN ensembles are considered when the networks are fed by two different time series for the anthropogenic RF. Namely, we refer to the anthropogenic forcing factors used in CMIP5 runs^20^ (RFANTHCMIP5) and in a notable work by Hansen *et al*.^21^ (RFANTHANSEN). We can consider the first time series as an upper limit for the RF of anthropogenic origin, while the second one represents a more similar estimation of anthropogenic forcings if compared with our original one. In any case, in both new time series the cooling role of sulphates is clearly less important, especially during the 1945–1975 hiatus period (see Supplementary Fig. S5).

Two new NN ensemble runs are performed through networks with architecture 3-4-1, as in the first numerical experiment presented in this paper. In these cases, the inputs are RFANTHCMIP5, RFSOLAR, RFVOL and RFANTHANSEN, RFSOLAR, RFVOL, respectively. The results of temperature reconstruction and attribution are shown in Supplementary Figs S6, 7 for the two cases.

When RFANTHCMIP5 is considered, NNs are still able to reconstruct the temperature data, including the recent global warming, in a satisfying manner (R = 0.912, RMSE = 0.109), even if the ensemble mean shows a continuous increasing trend since the end of the 19^th^ century and the 1945–1975 hiatus is not entirely caught: just a slowdown of warming can be appreciated in this period (see Supplementary Fig. S6a). This is obviously due to the decreased influence of the sulphates in these runs. The correspondent attribution run (Supplementary Fig. S6b) shows the same constant trend for the recent decades already found in the original run (Fig. 1b), but with lower values of temperature anomalies. This is perfectly understandable, because now the model for temperature reconstruction has been trained with higher values of anthropogenic forcings in the last decades and the difference in RFANTHCMIP5 between 1850 and the more recent years is higher in these runs.

When RFANTHANSEN is considered, the reconstruction performance is still quite satisfying (R = 0.909, RMSE = 0.111) and, once more, the 1945–1975 hiatus is not entirely caught (see Supplementary Fig. S7a). The attribution run shows a constant trend for the recent decades, which is very similar to that found in the original run (Fig. 1b).

Thus, the use of our adaptive method can lead to some considerations. First of all, the main attribution result, which claims the fundamental role of anthropogenic forcings in driving the recent global warming, seems robust: in any case of the anthropogenic RFs considered here, the attribution runs show the constancy of trend for recent decades, even if with slightly different T values in the case of RFANTHCMIP5.

Furthermore, in this section we find that considering other RFANTHs prevents a complete reconstruction of the 1945–1975 hiatus. Then a question may arise: what can be the role of natural variability in this framework? This is easily investigable by adopting the “residual strategy” already used in the first section, when the indices of variability are used in input to NNs in order to reconstruct the residual time series (observed T minus estimated T), or, more simply, by considering AMO as input together with external forcings.

In short, the results of these new runs show that the indices (and AMO in particular) lead to well reconstruct the cited hiatus even when RFANTHCMIP5 and RFANTHANSEN are considered. In the NN adaptive models adopted here, AMO is able to contribute substantially to the hiatus reconstruction, as sulphates mainly did in the standard runs with RFANTH. This is clearly visible in Supplementary Figs S8, 9: in particular, note that the 1945–1975 hiatus is not caught at all when AMO is kept constant.

Thus, in our NN runs, the relative weight of sulphates and AMO in determining the hiatus period depends on the choice of the anthropogenic forcing (and the cooling part – sulphates – inside it), so that no final conclusion can be drawn on which driver (sulphates or AMO) is the most influential on the hiatus temperatures. In the final Section we will examine again this problem.

### Nonlinear dependences on the forcings

The adoption of a fully nonlinear method in this research opens the possibility to investigate the nature of the dependence of temperature on forcings. In particular, it is interesting to explore how much the temperature response to high or low forcings is linear or nonlinear. The previous sensitivity study on forcing uncertainties can be therefore the framework for such kind of analysis.

We choose to limit our analysis to periods in which anthropogenic forcings and temperature are undoubtedly increasing, i.e. since 1976, and consider the reconstruction performance of both NN models (through their ensemble mean) and multilinear regressions when anthropogenic forcings are estimated as RFANTH, RFANTHCMIP5 and RFANTHANSEN. The results in terms of R are presented in Table 1.

**Table 1:** Sensitivity runs.

Anthropogenic RF	NN performance	Multilinear performance
RFANTHCMIP5	0.895	0.899
RFANTH	0.901	0.888
RFANTHANSEN	0.884	0.833

Performance of temperature reconstruction in terms of R on the recent global warming period (1976–2011) when the anthropogenic forcing is highly increased (RFANTHCMIP5) or is more similar (RFANTHANSEN) to our original estimate (RFANTH).

The situation seems quite clear. The NNs are able to reconstruct T much better than the multilinear regression when the forcing is low, while the performance are very similar when the forcing is high. This suggests a quasi-linear dependence of T on forcings for strong forcings and a more nonlinear behaviour for weak ones. At the present stage of investigation, we can only suppose that these stronger nonlinearities for low forcings could be linked to feedbacks or buffering effects (for instance, of the ocean). Further research seems to be needed on this topic.

### Possible time lags

Until now, in the present paper, synchronous time series are always considered. However, delayed time series could be analysed to investigate if some influence shows lags. It is well known that, at a monthly time resolution, many lags have been identified between forcing factors/natural variability and global T^22^. Vice versa, at annual time resolution, it has been shown that the indices of natural variability used in this paper do not present delayed influences on T^23^. Annual lags are usually considered just for RFANTH^22^. Thus, we have performed NN ensemble runs by considering a record (RFANTHLAG10) to be synchronized in our NN models with RFNAT, RFVOL and T related to ten years later.

The results obtained by this new set of inputs lead to a decrease in reconstruction performance and the analysis of the reconstructed time series shows a delay (estimated in about 6–8 years) in the starting point of the 1945–1975 hiatus and in the beginning of the recent global warming. In short, our method does not show an increase in performance when time lags are considered, in accordance with recent results that clearly show a time delay in the response of global temperature to emissions, but not to concentrations and the related radiative forcings^24^.

### Can sulphates drive AMO

In a previous Section we analysed the importance of sulphates and AMO as drivers of the temperatures during the 1945–1975 hiatus period, implicitly assuming that they represent independent influence factors on T. However, recently two papers investigated the role of sulphate aerosols in driving the North Atlantic climate variability^25,26^. In particular, the authors tried to answer this question: are sulphate aerosols a driver for this variability? The two papers achieved contrasting results. In this final section we can check how our approach could contribute to this debate.

To investigate the possible influence of RFSOX on AMO we built NN models (4-4-1 networks) in which we considered AMO as target and the following forcings as inputs: the natural forcings (RFSOLAR and RFVOL), the warming part of the anthropogenic forcing (RFWARM = RFGHG + RFBC) and its cooling part (RFSOX), so that the specific influence of sulphates in determining AMO can be investigated within the attribution runs.

**Figure 6:**
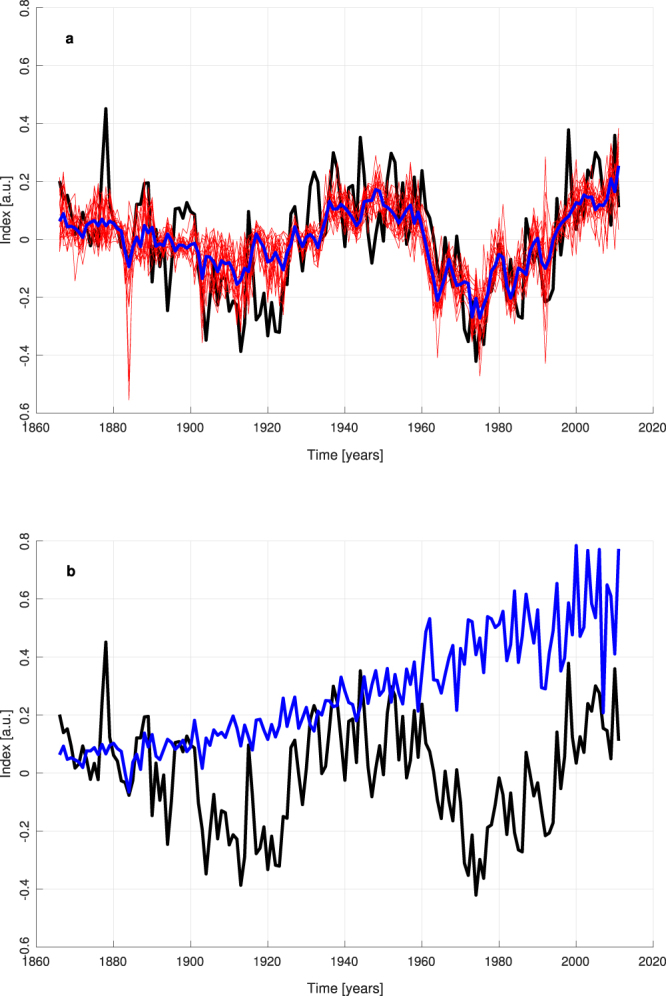
Reconstruction of AMO by NN models. Black line = observed AMO, red lines = results of ensemble runs, blue line = ensemble mean. (**a**) With real values of RFWARM, RFSOX, RFSOLAR and RFVOL as inputs. (**b**) Ensemble mean of attribution runs when RFSOX is fixed at its value of 1866.

The reconstruction performance of the AMO time series is quite successful (R = 0.677 and RMSE = 0.135 for or the ensemble mean): see Fig. 6a. In the framework of these NN validated models, the most notable result is obtained from the attribution runs performed by keeping RFSOX constant at its annual value of 1866. As shown in Fig. 6b, when RFSOX is constant, the shape of AMO is no more caught and a linear increase of the AMO index is expected by the ensemble mean of the models. This output is in agreement with the results by Booth *et al*.^25^, who found a similar shape when trying to reconstruct SSTs in the Atlantic region by keeping constant the aerosol forcing. This suggests that RFSOX can be an effective driver for AMO.

The results by Booth *et al*. have been criticized by Zhang *et al*.^26^ inside the framework of dynamical modelling, especially by considerations about salinity and ocean heat content. Unfortunately, the time series available for these variables are too short to be analysed in the context of NN modelling. However, we can perform further runs adopting the other forcing factors already used in the previous sensitivity studies to see if our result is robust.

After having split RFANTHCMIP5 and RFANTHANSEN into their warming and cooling parts (RFWARMCMIP5, RFSOXCMIP5, RFWARMHANSEN, RFSOXHANSEN), we repeated the previous experiment of AMO reconstruction by means of 4-4-1 NNs supplied with these new forcing series. Even if the reconstructions are now slightly less accurate, the attribution results when the cooling RF is fixed constant show that the shape of AMO is not caught at all also in these cases: the reconstruction shows a less increasing trend than in the run with our original forcings, but still quasi-linear (see Supplementary Figs S10, 11).

Thus, our results suggest that sulphates can be drivers for AMO, even if, of course, disentangling their complex relationship (and their mutual influence on T) probably would require more research.

## Conclusions

Our investigation by NNs shows that the attribution results by GCMs about the main role of the anthropic forcing are robust and reliable. This, added to our new results on the role of Sun (irrelevant for the trend of the last decades, and quite strong at the middle of the 20^th^ century) and the role of sulphates and natural variability, gives a more complete picture in attribution studies.

The sensitivity analyses performed here furtherly show that the NN attribution method itself can be considered robust with reference to uncertainties in anthropogenic forcings. Moreover, strong forcings seem to act in a quasi-linear manner, while the relationship between weaker forcings and temperature appears to be more nonlinear. At our annual time resolution, no delayed influence of forcings on temperature is clearly evident. Finally, our work shows that sulphates and AMO both contribute in driving the temperature behaviour (especially in the 1945–1975 hiatus period), and the NN method adopted here permits also to establish a potential driving role of sulphates on AMO which deserves further investigation.

Incidentally, our work can be relevant also for the climate debate on the media, where the anthropogenic influence on the global warming is often denied, basing on the critics to GCMs and their attribution results.

## Methods

### NN tool

The NNs used in this paper are quite standard feedforward networks with backpropagation training and one hidden layer, which allows them to be “universal approximators”^27,28^. These NNs are endowed with hyperbolic-tangent transfer functions at the hidden level and a linear function at the output neuron.

A training-validation-test procedure must be applied for obtaining a nonlinear relationship between inputs and targets (to be approximated by outputs) which can be generally valid. In particular, the connection weights (the free parameters of the NNs) have to be fixed on the training set – by stopping the training phase when the error on the validation set begins to increase – and the generalization performance must be measured using a third set (unknown to the NNs), the test set. The tool^8^ adopted in this paper allows us to maximize the members of the training set, while leaving good generalization performance.

In practice, starting from the first year of the dataset, a single input-target pattern is extracted from the total available dataset and considered as test set. A validation set is then randomly chosen (it represents the 10% of patterns) and the remaining patterns constitute the training set. After the run of the NN model, the connection weights are fixed, a transfer function is found from inputs to output and an estimation for the first value of T is obtained.

However, this result of reconstruction can be influenced by the specific random choice of the initial weights and the members of the validation set. Thus, multiple runs can be performed by choosing other random values for initial weights and members for the validation set. We perform 20 runs in an ensemble approach and then calculate the average of the reconstruction outputs, with the same rationale adopted in ensemble runs of GCMs when one considers the ensemble mean.

After this reconstruction of the first annual value of T, we follow the same procedure for the other years (each of them becomes – sequentially – the test set), thus permitting the estimation of all output values (and ensemble means) at the end of the procedure.

The training-validation-test procedure is not the only mean to avoid overfitting problems. Here, we keep very low the number of inputs and hidden neurons, so that the free parameters of our NN models are an order of magnitude less than the number of patterns in the training set.

### Code availability

MATLAB® code for NN generalized leave-one-out modelling and attribution runs is included in the Supplementary information.

### Data availability

We consider annual data of global combined land and marine temperature anomalies since 1850 (HadCRUT4)^29^ from the Met Office Hadley Centre, available at https://crudata.uea.ac.uk/cru/data/temperature/.

As far as data about radiative forcings are concerned, we use the freely available dataset collected at http://www.sterndavidi.com/datasite.html and referred to a paper by Stern & Kaufmann^30^, where the data on GHGs concentrations are taken from the NASA/GISS website and the related RF calculations are performed through standard formulas^31,32^.

As for sulphates, data about global estimates of their emissions^33,34^ are available only till 2011. The calculation^30^ of direct and indirect RFs are based on slight modifications of previous studies^35,36^. Data about RF of black carbon^37^ come from the RCP8.5 scenario.

Solar irradiance is approximated by an index previously assembled^38^ and available at https://data.giss.nasa.gov/modelforce/solar.irradiance/. The conversion from solar irradiance to RFSOLAR is obtained in a standard way^32^.

Volcanic activity of dust emission is considered through the optical thickness data^39^ from https://data.giss.nasa.gov/modelforce/strataer/ and RFVOL is −27 times the optical thickness^30^.

In this paper we consider three indices for representing patterns of natural variability, i.e.:

- AMO^40^ since 1856: data available at www.esrl.noaa.gov/psd/data/timeseries/AMO;
- PDO^41^ since 1854: data available at https://www.ncdc.noaa.gov/teleconnections/pdo/;
- SOI, related to ENSO^42–44^ since 1866: data available at www.cru.uea.ac.uk/cru/data/soi/soi.dat.

Data about anthropogenic radiative forcings in the alternative scenarios used for the sensitivity analyses are available at https://data.giss.nasa.gov/modelforce/Fi_Miller_et_al14_upd.txt and https://data.giss.nasa.gov/modelforce/Fe_H11_1880-2011.txt, for CMIP5^20^ and Hansen *et al*.^21^, respectively. The first available year for the latter dataset is 1880, so that here it is extended backward to 1850 by filling the first 30 years with zero values for anthropogenic RF.

The dataset used in this paper is included in the Supplementary information.

## Electronic supplementary material

Supplementary information
