# Supplementary material for: Attribution of recent temperature behaviour reassessed by a neural-network method
Source: Sci Rep. 2017 Dec 15;7:17681. doi: 10.1038/s41598-017-18011-8 (PMC5732275; doi:10.1038/s41598-017-18011-8)
Supplement: Supplementary file 1 — Supplementary information [file 41598_2017_18011_MOESM1_ESM.pdf]

Supplementary information

of

Attribution of recent temperature behaviour reassessed by a neural-network method

Antonello Pasini[1], Paolo Racca[2], Stefano Amendola[3], Giorgio Cartocci[3] & Claudio Cassardo[4,5]

[1]Institute of Atmospheric Pollution Research, National Research Council, Rome, Italy
[2]Department of Economics and Statistics, University of Turin, Torino, Italy
[3]Department of Mathematics and Physics, Roma Tre University, Rome, Italy
[4]Department of Physics, University of Turin, Torino, Italy
[5]Department of Atmospheric Science and Engineering, Ewha Womans University, Seoul, Korea

Supplementary figures

Supplementary table

NN Code

Dataset of main runs

Dataset of sensitivity runs

Supplementary figures

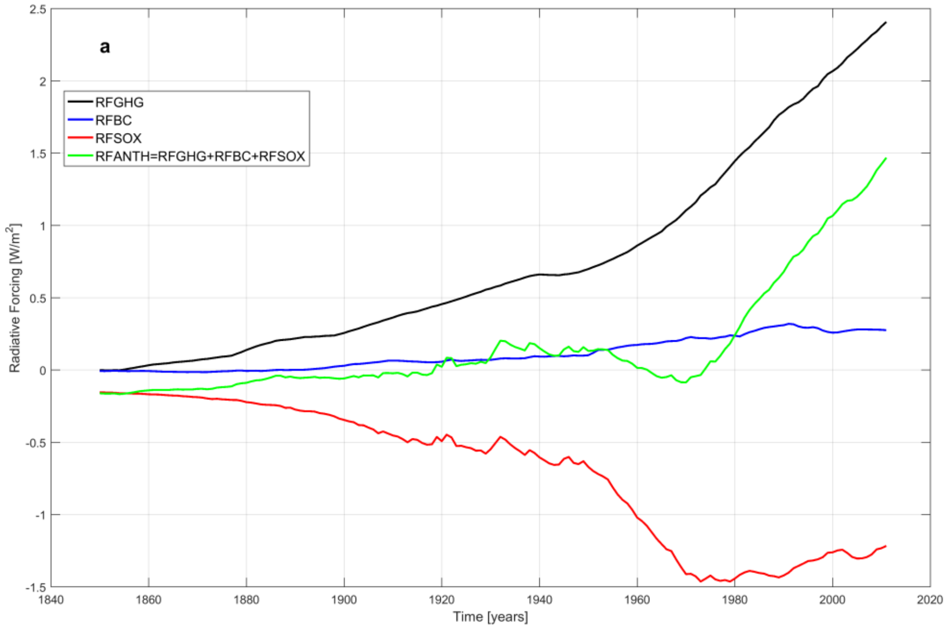

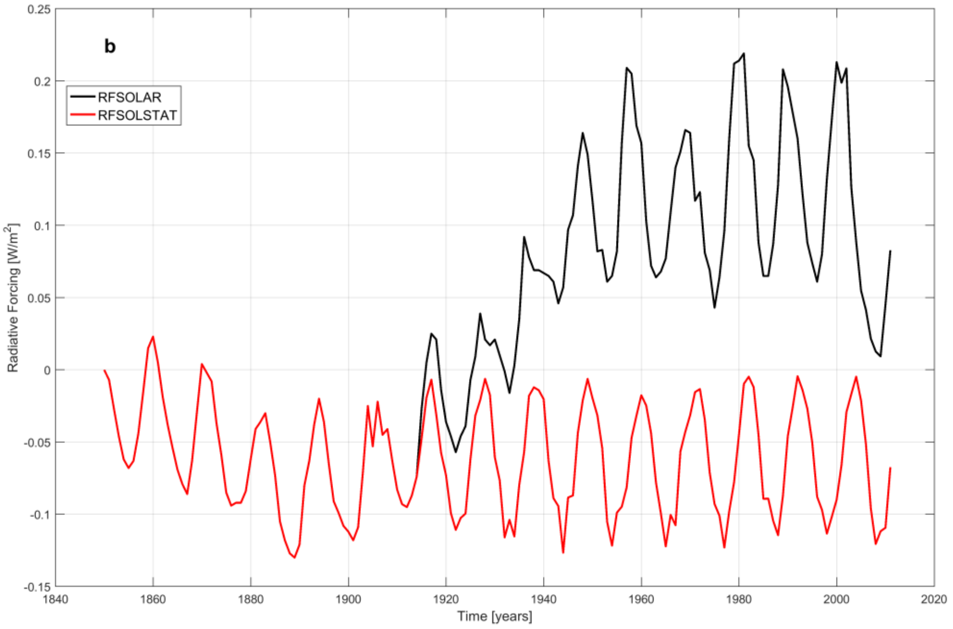

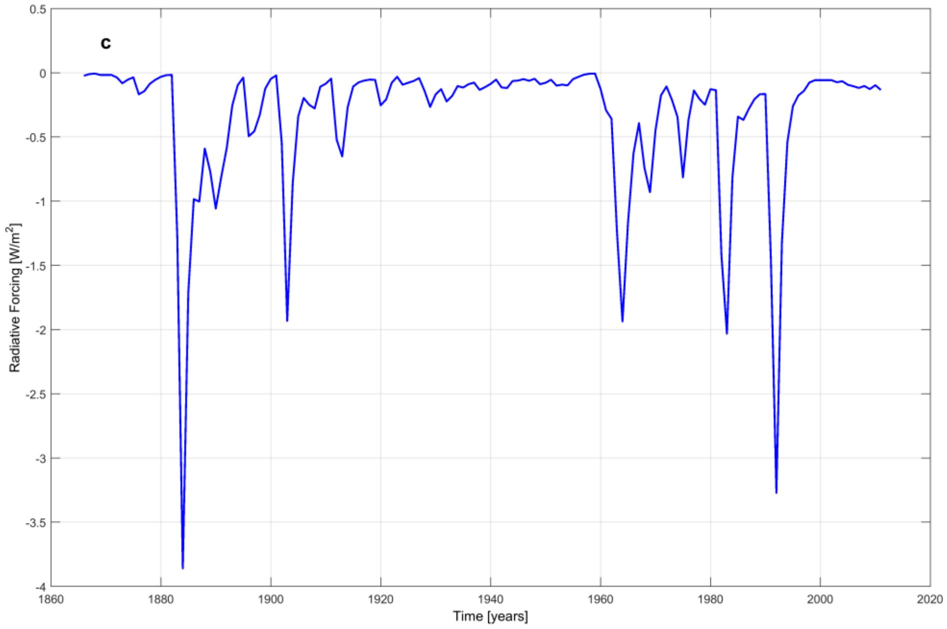

Figure S1 | External forcings used as inputs for the NN models. **a,** Anthropogenic forcings anomalies. **b,** Observed solar radiative forcing anomalies (black line) and synthetic time series of the same forcing under the assumption of stationarity. **c,** Volcanic radiative forcing.

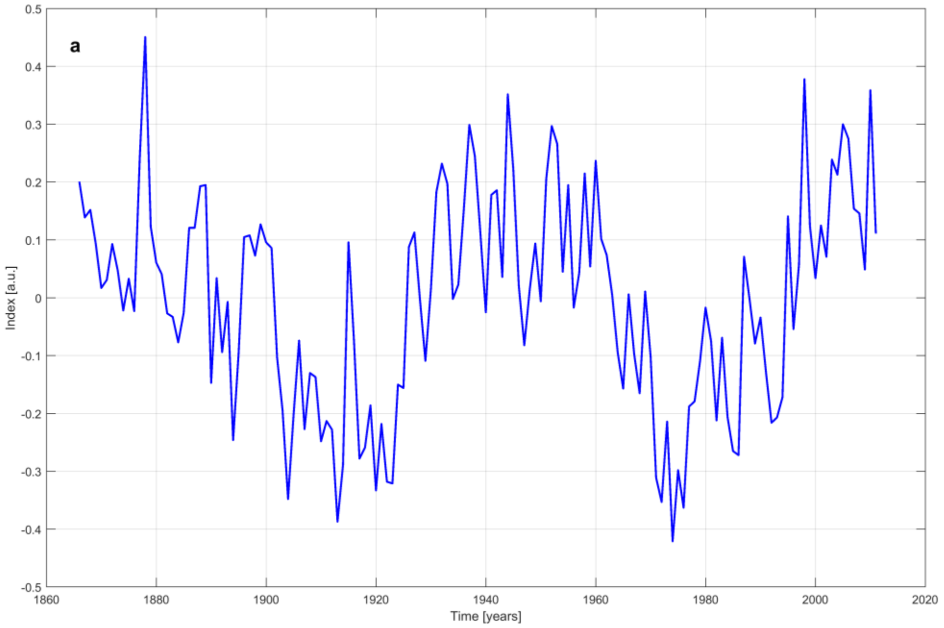
a
Index [a.u.]
Time [years]
0.5
0.4
0.3
0.2
0.1
0
-0.1
-0.2
-0.3
-0.4
-0.5
1860
1880
1900
1920
1940
1960
1980
2000
2020

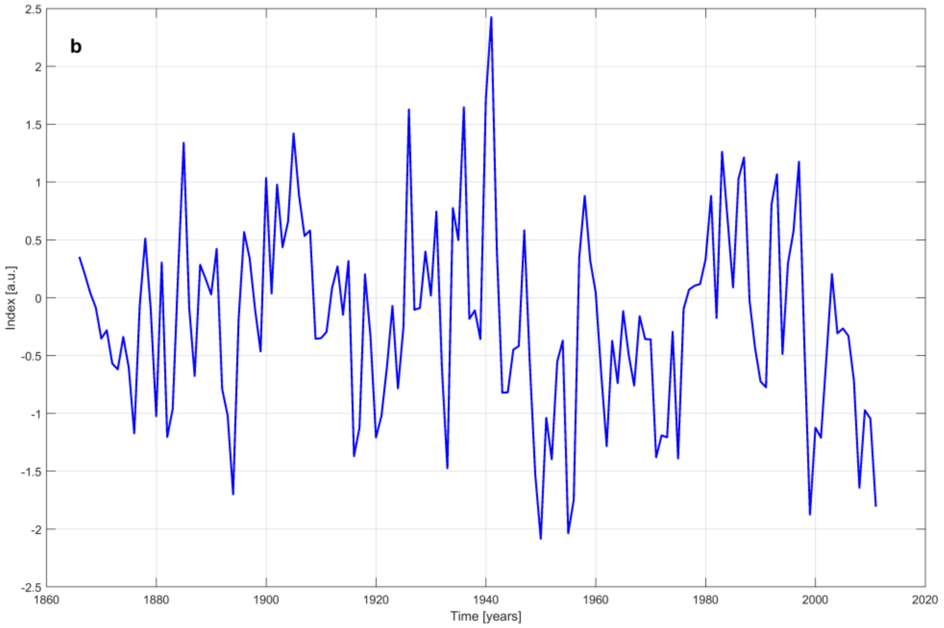
b
Index [a.u.]
Time [years]
2.5
2
1.5
1
0.5
0
-0.5
-1
-1.5
-2
-2.5
1860
1880
1900
1920
1940
1960
1980
2000
2020

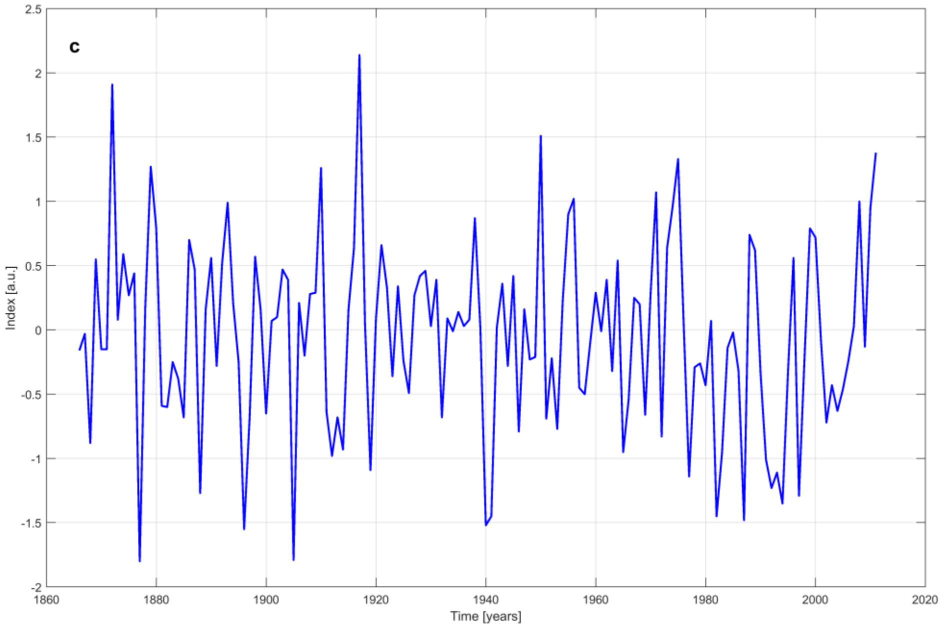

Figure S2 | Indices of natural variability considered in this paper. **a,** AMO. **b,** PDO. **c,** SOI.

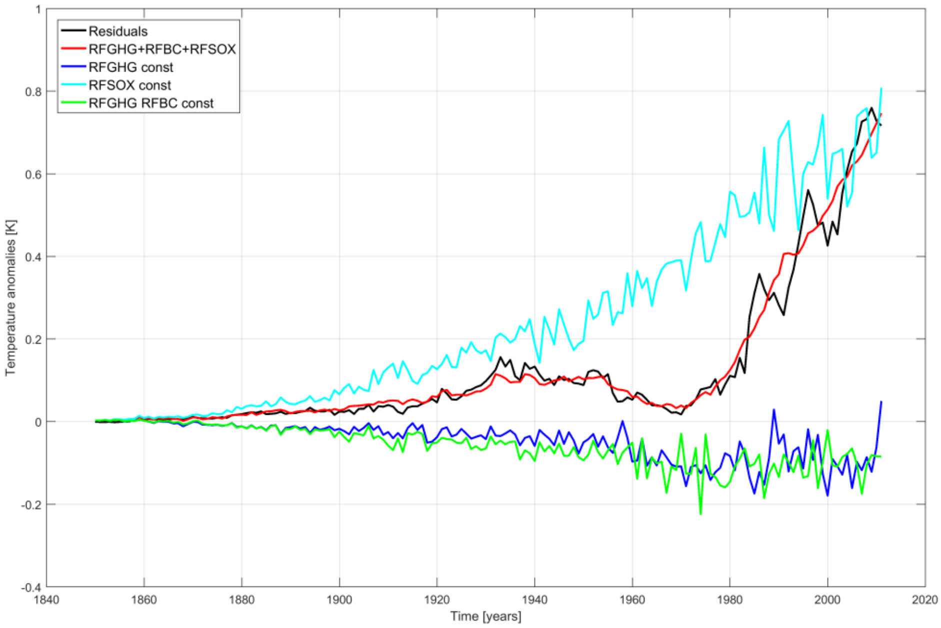

Figure S3 | Reconstruction and attribution experiments on the residuals which represent the influence of the total anthropogenic RF on T. Black line = identified residuals, red line = reconstructed residuals by all inputs with actual values, blue, light blue and green lines = attribution runs with constant forcing(s).

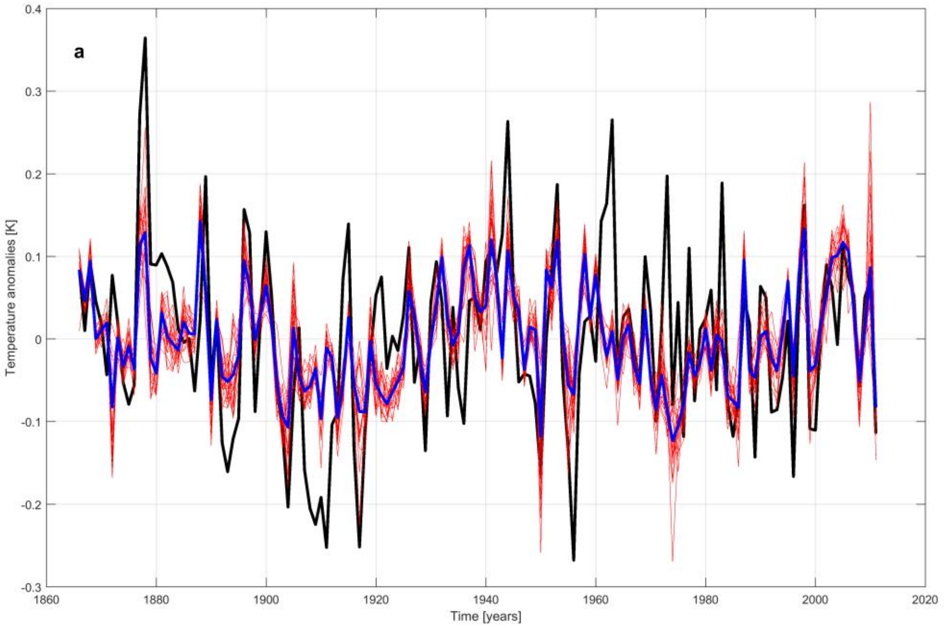
a
Temperature anomalies [K]
Time [years]

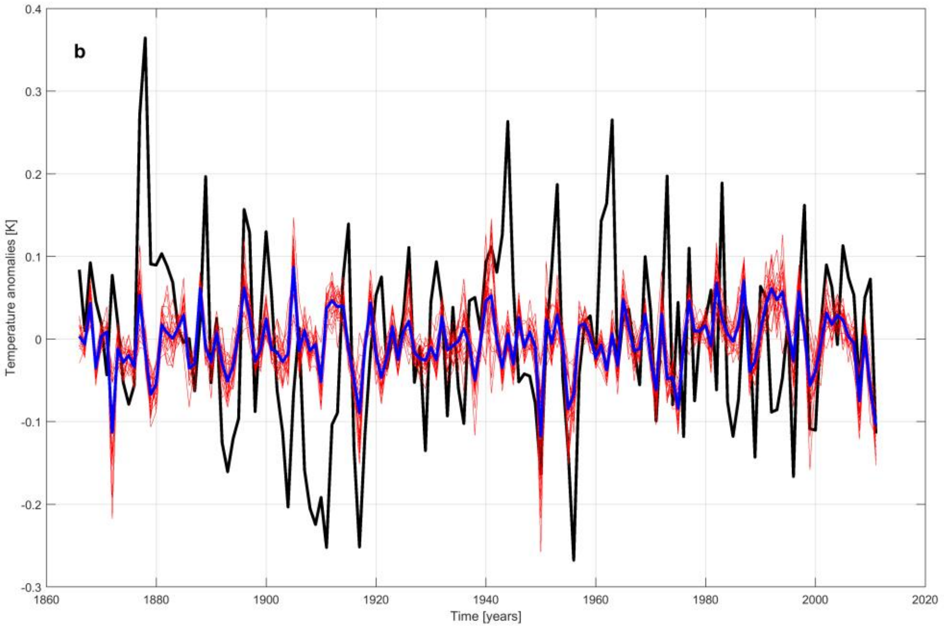
b
Temperature anomalies [K]
Time [years]

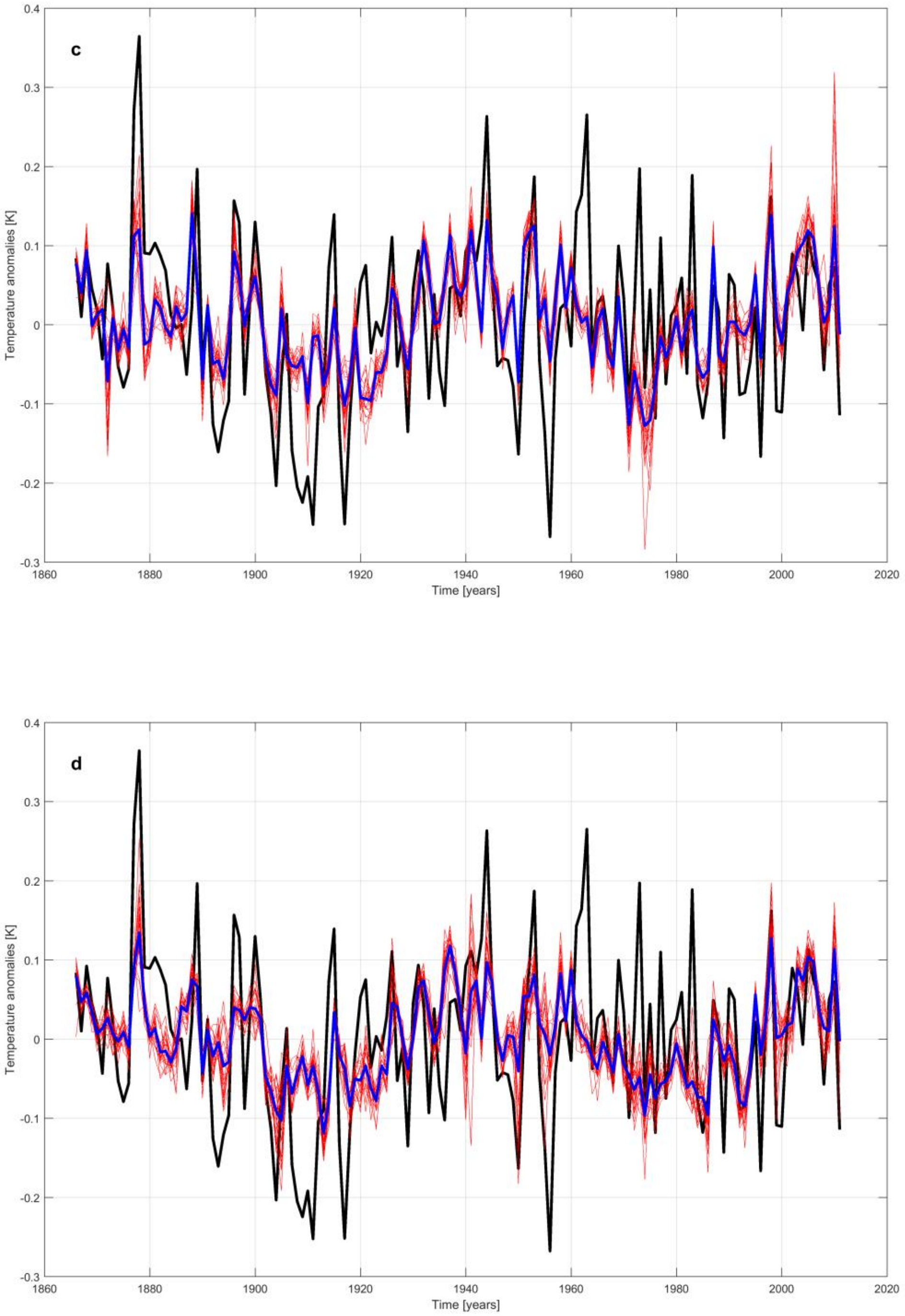

Figure S4 | Results about the role of circulation patterns on residual variability not caught by external forcings. **a,** Reconstruction of the residual series by NNs endowed with AMO, SOI and PDO as inputs. **b-d,** Attribution experiments in which are kept constant: AMO (b), PDO (c), SOI (d).

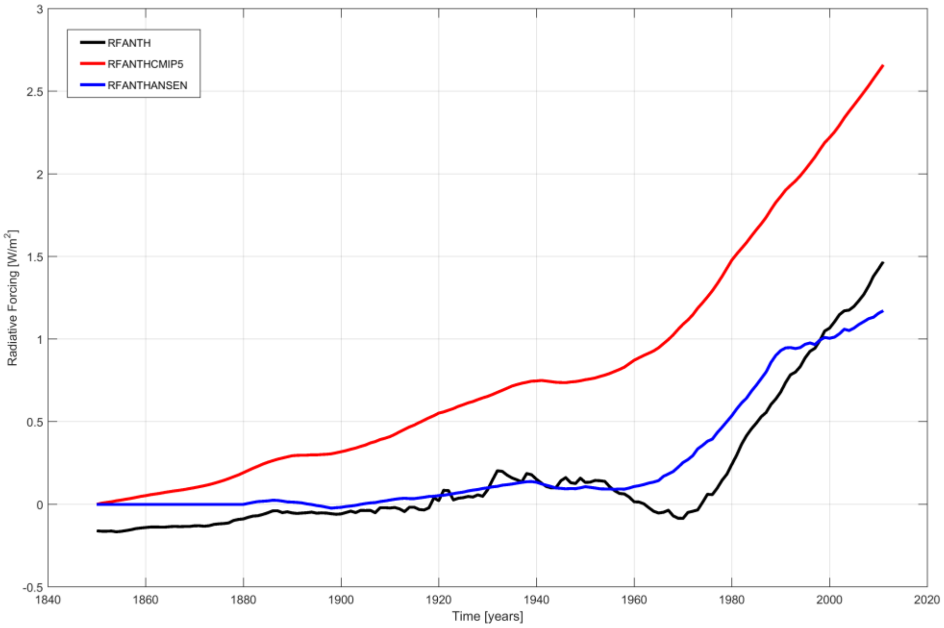

Figure S5 | Anthropogenic forcings used as inputs in the NN sensitivity runs.

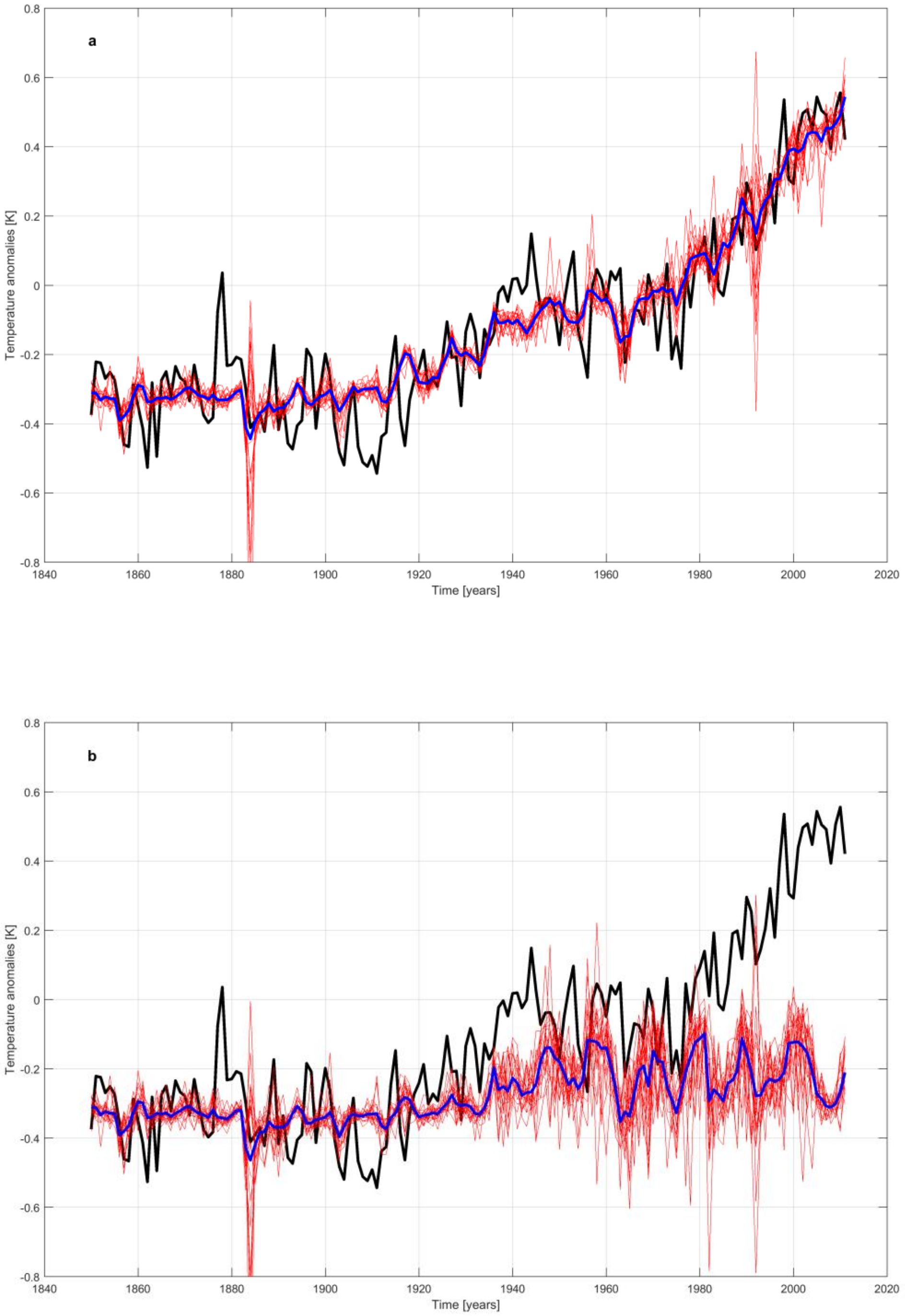

Figure S6 | Reconstruction of global T by NN models with CMIP5 anthropogenic forcing. Black line = observed T, red lines = results of ensemble runs, blue line = ensemble mean. **a,** With real values of RFANTHCMIP5, RFSOLAR and RFVOL as inputs. **b,** Attribution runs when RFANTHCMIP5 is fixed at its value of 1850.

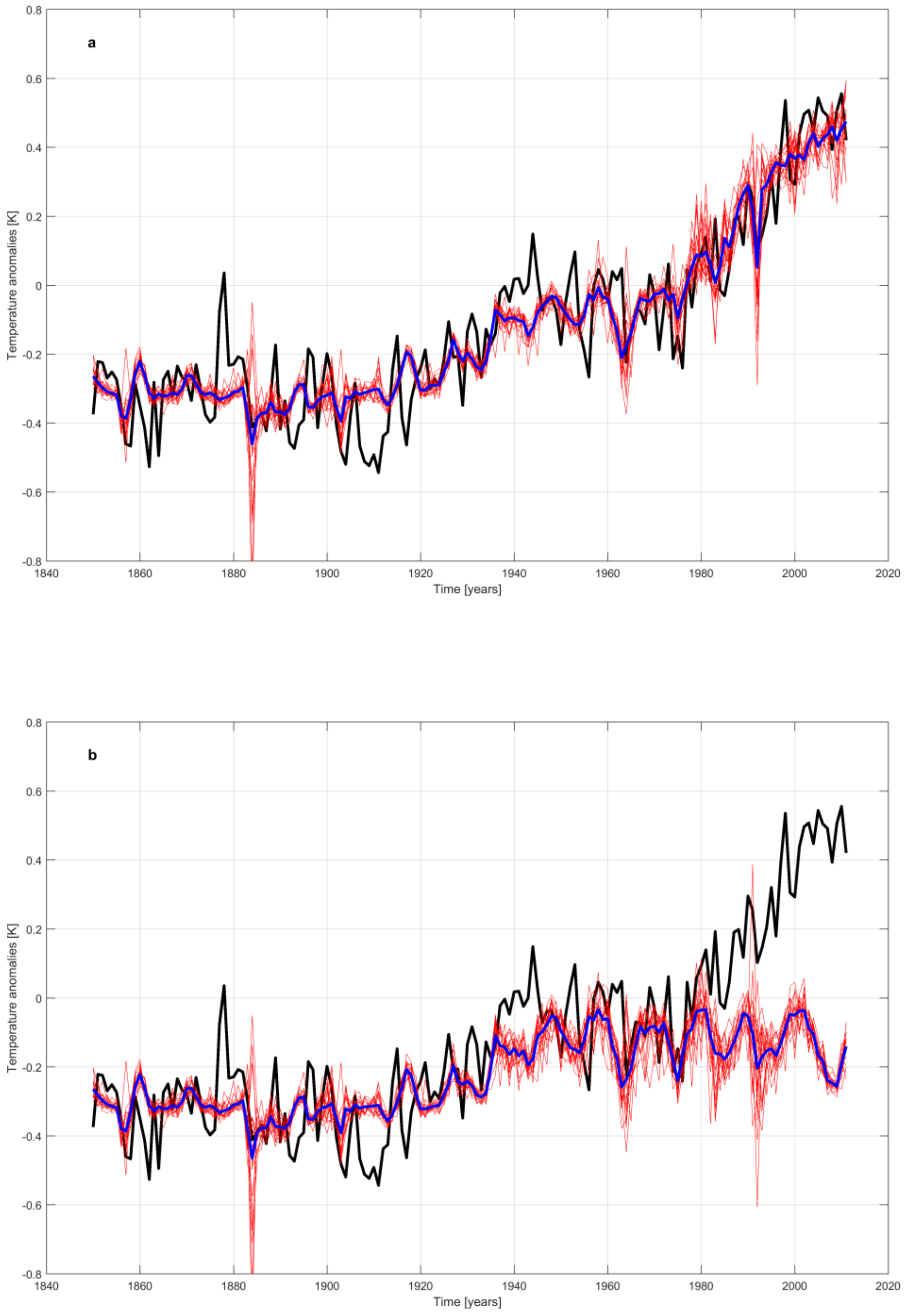

Figure S7 | Reconstruction of global T by NN models with anthropogenic forcing by Hansen et al. Black line = observed T, red lines = results of ensemble runs, blue line = ensemble mean. **a,** With real values of RFANTHANSEN, RFSOLAR and RFVOL as inputs. **b,** Attribution runs when RFANTHANSEN is fixed at its value of 1850.

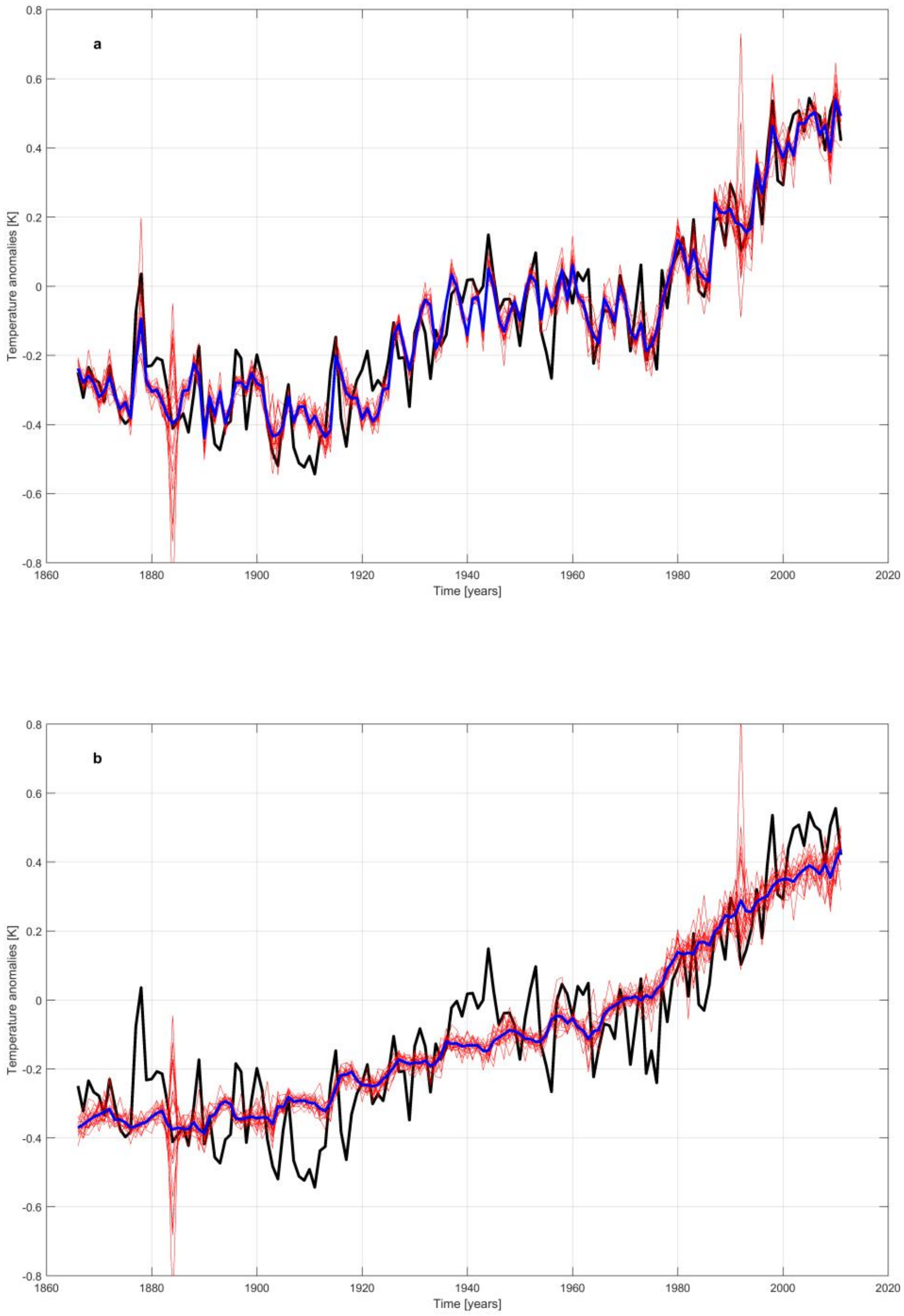

Figure S8 | Reconstruction of global T by NN models with CMIP5 anthropogenic forcing and AMO. Black line = observed T, red lines = results of ensemble runs, blue line = ensemble mean. **a,** With real values of RFANTHCMIP5, RFSOLAR, RFVOL and AMO as inputs. **b,** Attribution runs when AMO is kept fixed at its mean value.

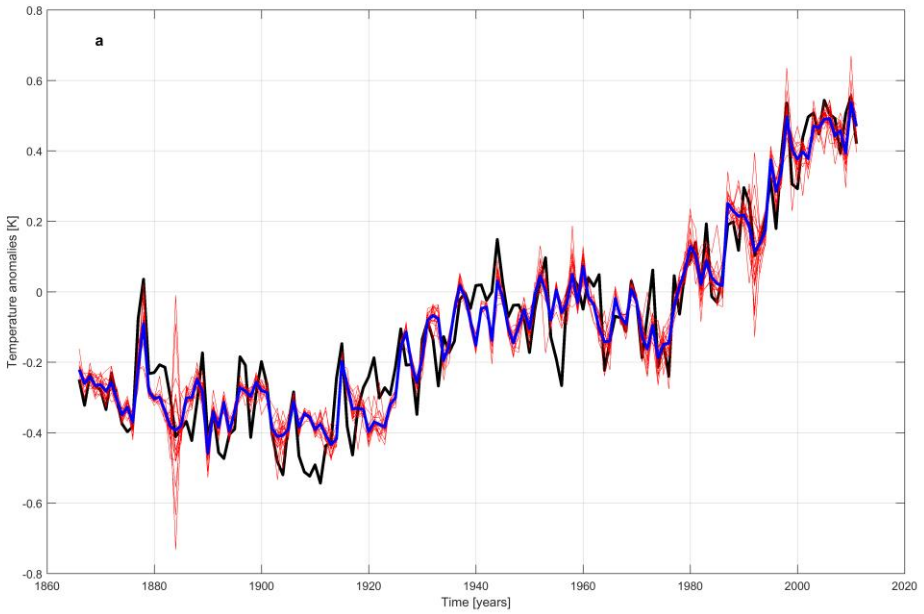

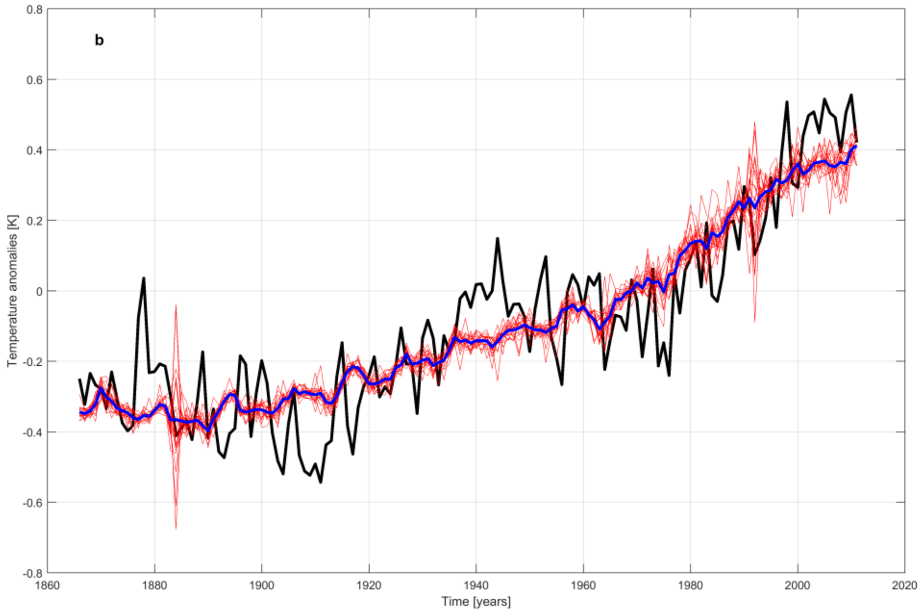

Figure S9 | Reconstruction of global T by NN models with anthropogenic forcing by Hansen et al. and AMO. Black line = observed T, red lines = results of ensemble runs, blue line = ensemble mean. **a,** With real values of RFANTHANSEN, RFSOLAR, RFVOL and AMO as inputs. **b,** Attribution runs when AMO is kept fixed at its mean value.

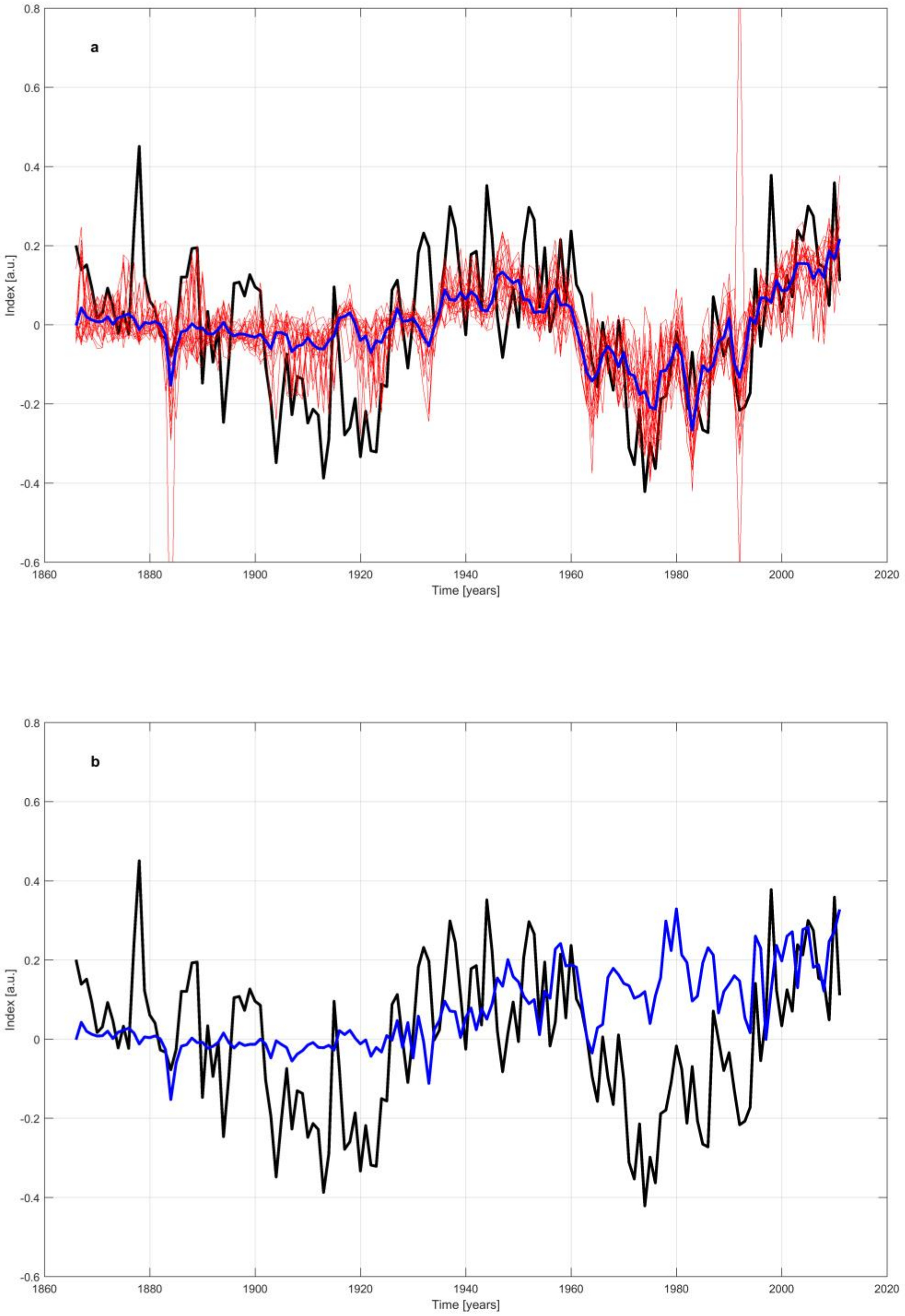

Figure S10 | ** Reconstruction of AMO by NN models. Black line = observed AMO, red lines = results of ensemble runs, blue line = ensemble mean. **a, With real values of RFWARMCMIP5, RFSOXCMIP5, RFSOLAR and RFVOL as inputs. **b,** Attribution runs when RFSOXCMIP5 is fixed at its value of 1866.

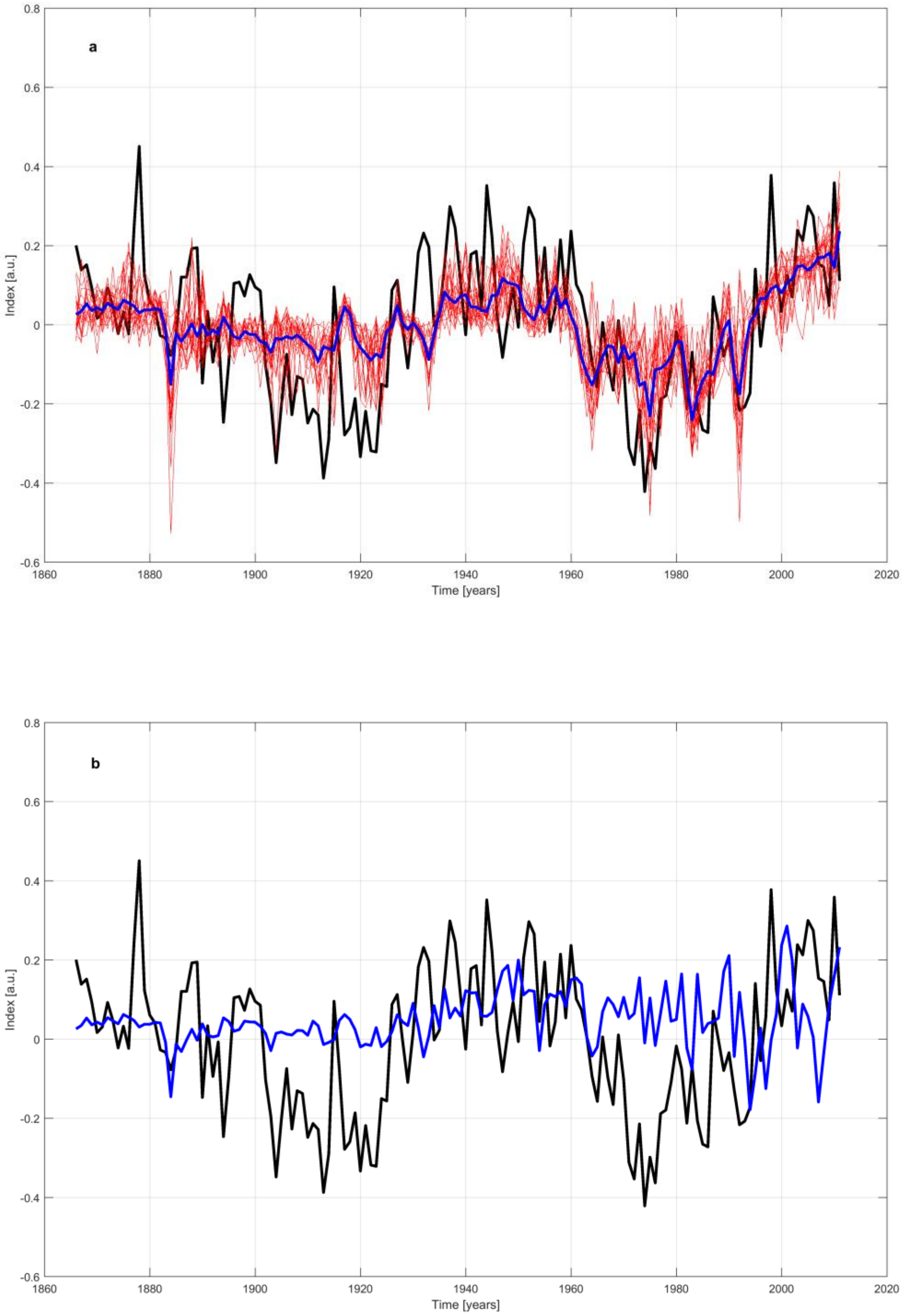

Figure S11 | Reconstruction of AMO by NN models. Black line = observed AMO, red lines = results of ensemble runs, blue line = ensemble mean. **a,** With real values of RFWARMHANSEN, RFSOXHANSEN, RFSOLAR and RFVOL as inputs. **b,** Attribution runs when RFSOXHANSEN is fixed at its value of 1866.

Supplementary table

Table S1 | Performance of reconstruction and attribution NN runs.

NN mode	Validation inputs/Attribution runs	R	RMSE (K)
Reconstruction	RFANTH, RFSOLAR, RFVOL	0.913	0.109
Attribution	Constant RFANTH	0.645	0.247
Attribution	RFSOLSTAT	0.836	0.177
Attribution	Constant RFVOL	0.909	0.113
Reconstruction	Constant RFANTH + residuals from RFGHG, RFBC, RFSOX	0.905	0.113
Attribution	Constant RFANTH + constant RFGHG in residual run	0.489	0.291
Attribution	Constant RFANTH + constant RFGHG, RFBC in residual run	0.492	0.299
Attribution	Constant RFANTH + constant RFSOX in residual run	0.848	0.201
Reconstruction	RFANTH, RFSOLAR, RFVOL + residuals from AMO, SOI, PDO	0.944	0.089
Attribution	Constant AMO in residual run	0.918	0.106
Attribution	Constant SOI in residual run	0.937	0.094
Attribution	Constant PDO in residual run	0.941	0.092

NN code

```
% Script for NN attribution studies.
% In this configuration it works using RFANTH, RFSOLAR, RFVOL as
% predictors
% for mean global temperature. Also attribution is performed
% using during
% test of neural network three different configurations:
% - RFANTH kept constant at 1850 value;
% - RFSOLSTAT
% - RFVOL = 1


clear
close all
clc

dataset = xlsread('dataset_articolo.xls','Foglio2');

% Matrix "dataset" content (data from 1850):
% 1 Time (years)
% 2 HadCRUT4 Global Mean Temperature Anomalies
% 3 RFGHG: Radiative Forcing anomalies of Greenhouse Gases
% 4 RFBC: Radiative Forcing anomalies of Black Carbon
% 5 RFSOX: Radiative Forcing anomalies of sulfates
% 6 RFSOLAR: Radiative Forcing anomalies of Total Solar
% Irradiance
% 7 RFVOL: Radiative forcing of stratospheric sulfates
% (Volcanoes)
% 8 AMO: Atlantic Multidecadal Oscillation (starting from 1866)
% 9 SOI: Southern Oscillation Index (starting from 1866)
% 10 PDO: Pacific Decadal Oscillation (starting from 1866)
% 11 RFANTH: Total anthropogenic radiative forcing (RFGHG + RFBC
% + RFSOX)
% 12 RFANTHMIN: Minimum total anthropogenic radiative forcing
% (RFGHG +   %1.5*RFSOX)
% 13 RFANTHMAX: Maximum total anthropogenic radiative forcing
% (RFGHG + %3*RFBC + 0.5*RFSOX)
% 14 RFSOLSTAT: Reconstructed Solar Radiative Forcing anomalies
% on the %basis of first 65 values of RFSOLAR series

%% PRELIMINARY SETTINGS ----------------------------------------
--------
nval = 15; % Number of elements of validation set
EnsNum = 20;  % Number of ensemble members

vettColIN = [11 6 7]; % Input elements position
vettColInAT = [11 14 7]; % Input elements position for
% attribution test
colDiff = find(vettColIN~=vettColInAT);
vettColTG = 2; % Target elements position
Ntarget = length(vettColTG); % Number of target
```

```
Npattern = length(dataset); % Length of dataset

neurons = 4; % Number of neurons of hidden layer
max_ep = 5000;      % Maximum number of training epochs
min_mse = 0.;     % Minimum mse for training
goodperf = 0.160;  % Minimum accepted performance
learningRate = 0.35;
momentum = 0.6;
% ---------------------------------------------------------------
-------

%% MATRICES DEFINITION -----------------------------------------
---------
inputMatr = (dataset(:,vettColIN))';
inputMatrATTR = (dataset(:,vettColInAT))';
targetMatr = (dataset(:,vettColTG))';

% Basic test
matrensemble1 = zeros(Ntarget,Npattern,EnsNum);
% RFANTH at levels of 1850 - used during test of neural network
matrensemble2 = zeros(Ntarget,Npattern,EnsNum);
% RFSOLSTAT - used during test of neural network
matrensemble3 = zeros(Ntarget,Npattern,EnsNum);
% RFVOL=1 - used during test of neural network
matrensemble4 = zeros(Ntarget,Npattern,EnsNum);

y = zeros(size(targetMatr));
ySENS2 = zeros(size(targetMatr));
ySENS3 = zeros(size(targetMatr));
ySENS4 = zeros(size(targetMatr));

ynorm = zeros(size(targetMatr));
ynormSENS2 = zeros(size(targetMatr));
ynormSENS3 = zeros(size(targetMatr));
ynormSENS4 = zeros(size(targetMatr));
% ---------------------------------------------------------------
-------

%% MATRICES NORMALIZATION --------------------------------------
---------
% Used for training
inputMatrNorm = mapminmax(inputMatr);
[targetMatrNorm,targetMatrS] = mapminmax(targetMatr);

% Used for test - RFANTH at levels of 1850
inputMatrNorm_TEST2 = mapminmax(inputMatr);
% Used for TEST - RFSOLSTAT
inputMatrNorm_TEST3 = mapminmax(inputMatrATTR);
% Used for TEST - RFVOL=1
inputMatrNorm_TEST4 = mapminmax(inputMatr);

%% Fix same values for attribution test
% Used for TEST - RFANTH kept constant at levels of 1850
for i = 1:Npattern
```

```
    index = find(vettColIN==11);
    inputMatrNorm_TEST2(index,i) = inputMatrNorm_TEST2(index,1);
end

% Used for TEST - RFSOLSTAT
for k = 1:length(colDiff)
    [max2, indMax] = max(inputMatrATTR(colDiff(k),1:65));
    [min2, indMin] = min(inputMatrATTR(colDiff(k),1:65));

    estSup = inputMatrNorm(colDiff(k),indMax);
    estInf = inputMatrNorm(colDiff(k),indMin);
    inputMatrNorm_TEST3(colDiff(k),:) = mapminmax
(inputMatrATTR(colDiff(k),:), estInf, estSup);
end

% Used for TEST - RFVOL=1
ColNatCost = find(vettColIN==7);
for i=1:Npattern
    inputMatrNorm_TEST4(ColNatCost,i) = 1;
end
% ---------------------------------------------------------------
-------

%% Neural network ----------------------------------------------
-------
net = newff(inputMatrNorm,targetMatrNorm,neurons, {'tansig'
'purelin'}, 'traingdm');
% ---------------------------------------------------------------
-------


%% Figure settings ---------------------------------------------
-------
% Figure 1 - Basic test
scrsz = get(0,'ScreenSize');
fig1 = figure('Position',[1 scrsz(2) scrsz(3) scrsz(4)]);
plot(dataset(:,1),targetMatr(1,:),'k','LineWidth',3);
hold on
grid on
title('Predictors: RFANTH - RFSOLAR - RFVOL','Fontsize',16)
ylabel('Temperature anomalies [K]')
xlabel('Time [Years]')

% Figure 2 - Attribution: RFANTH at levels of 1850
scrsz = get(0,'ScreenSize');
fig2 = figure('Position',[1 scrsz(2) scrsz(3) scrsz(4)]);
plot(dataset(:,1),targetMatr(1,:),'k','LineWidth',3);
hold on
grid on
title('Attribution: RFANTH const','Fontsize',16)
ylabel('Temperature anomalies [K]')
xlabel('Time [Years]')

% Figure 3 - Attribution: RFSOLSTAT
```

```
scrsz = get(0,'ScreenSize');
fig3 = figure('Position',[1 scrsz(2) scrsz(3) scrsz(4)]);
plot(dataset(:,1),targetMatr(1,:),'k','LineWidth',3);
hold on
grid on
title('Attribution: RFSOLSTAT const','Fontsize',16)
ylabel('Temperature anomalies [K]')
xlabel('Time [Years]')

% Figure 4 - Attribution: RFVOL=1
scrsz = get(0,'ScreenSize');
fig4 = figure('Position',[1 scrsz(2) scrsz(3) scrsz(4)]);
plot(dataset(:,1),targetMatr(1,:),'k','LineWidth',3);
hold on
grid on
title('Attribution: RFVOL=1','Fontsize',16)
ylabel('Temperature anomalies [K]')
xlabel('Time [Years]')
% ---------------------------------------------------------------
-------


%% NEURAL NETWORK TRAINING-VALIDATION-TEST ----------------------
-------
for numensemble = 1:EnsNum

    for i=1:Npattern

        if(i==1)
            TrValIND = (2:Npattern);
        elseif(i==Npattern)
            TrValIND = (1:Npattern-1);
        else
            TrValIND = [(1:i-1) (i+1:Npattern)];
        end

        [trainIND, valIND] = ValTrainDiv(TrValIND,nval);

        net.divideFcn = 'divideind';
        net.divideParam.trainInd = trainIND;
        net.divideParam.valInd = valIND;
        net.divideParam.testInd = i;

        net.performFcn = 'mse';
        net.trainParam.lr = learningRate;
        net.trainParam.showWindow=0;
        net.trainParam.show = 25;
        net.trainParam.max_fail = 20;
        net.trainParam.mc = momentum;
        net.trainParam.epochs = max_ep;
        net.trainParam.goal = min_mse;
        net.trainParam.min_grad = 0;
        contat=1;
```

```
        for k=1:100
           net = init(net);
           [net,tr] = train(net,inputMatrNorm,targetMatrNorm);

%If the epochs of convergence are lesser than 100, training is
performed %again
           if(tr.best_epoch < 100)
               continue;
           end

% If performance is lesser than minimum accepted performance
           if(tr.perf(1,tr.best_epoch+1)<=goodperf)

               % Basic Test
               ynorm(:,i) = sim(net,inputMatrNorm(:,i));
               y(:,i) =
mapminmax('reverse',ynorm(:,i),targetMatrS);

               % Attribution: RFANTH at levels of 1850
               ynormSENS2(:,i) =
sim(net,inputMatrNorm_TEST2(:,i));
            ySENS2(:,i) =
mapminmax('reverse',ynormSENS2(:,i),targetMatrS);

               % Attribution: RFSOLSTAT
               ynormSENS3(:,i) =
sim(net,inputMatrNorm_TEST3(:,i));
            ySENS3(:,i) =
mapminmax('reverse',ynormSENS3(:,i),targetMatrS);

               % Attribution: RFVOL=1
               ynormSENS4(:,i) =
sim(net,inputMatrNorm_TEST4(:,i));
            ySENS4(:,i) =
mapminmax('reverse',ynormSENS4(:,i),targetMatrS);

               break;

           else

           end

        end
    end

    % Plot of ensemble members
    figure(1)
    plot(dataset(:,1),y(1,:),'r');
    matrensemble1(:,:,numensemble)=y;

    figure(2)
    plot(dataset(:,1),ySENS2(1,:),'r');
    matrensemble2(:,:,numensemble)=ySENS2;

    figure(3)
```

```
    plot(dataset(:,1),ySENS3(1,:),'r');
    matrensemble3(:,:,numensemble)=ySENS3;

    figure(4)
    plot(dataset(:,1),ySENS4(1,:),'r');
    matrensemble4(:,:,numensemble)=ySENS4;

end
% -------------------------------------------------------------
-------


ENS_MEAN = mean(matrensemble1,3);
ENS_MEAN_SENS2 = mean(matrensemble2,3);
ENS_MEAN_SENS3 = mean(matrensemble3,3);
ENS_MEAN_SENS4 = mean(matrensemble4,3);
matrensemble1(1,:,21) = ENS_MEAN;
matrensemble2(1,:,21) = ENS_MEAN_SENS2;
matrensemble3(1,:,21) = ENS_MEAN_SENS3;
matrensemble4(1,:,21) = ENS_MEAN_SENS4;




% Plot of the ensemble mean
figure(1)
plot(dataset(:,1),ENS_MEAN,'b','LineWidth',3)
figure(2)
plot(dataset(:,1),ENS_MEAN_SENS2,'b','LineWidth',3)
figure(3)
plot(dataset(:,1),ENS_MEAN_SENS3,'b','LineWidth',3)
figure(4)
plot(dataset(:,1),ENS_MEAN_SENS4,'b','LineWidth',3)

% Save data
xlswrite('NeuralNetworkSimulations.xls',squeeze(matrensemble1),1
,'C2:W163')
xlswrite('NeuralNetworkSimulations.xls',squeeze(matrensemble2),2
,'C2:W163')
xlswrite('NeuralNetworkSimulations.xls',squeeze(matrensemble3),3
,'C2:W163')
xlswrite('NeuralNetworkSimulations.xls',squeeze(matrensemble4),4
,'C2:W163')


% Save images
saveas(fig1, 'Predictors_RFANTH_RFSOLAR_RFVOL.fig','fig');
saveas(fig2, 'Attribution_RFANTH_1850.fig','fig');
saveas(fig3, 'Attribution_RFSOLSTAT.fig','fig');
saveas(fig4, 'Attribution_RFVOL1.fig','fig');
```

Dataset of main runs

Year	HADCRUT4	RFGHG	RFBC	RFSOX	RFSOLAR	RFVOL	AMO	SOI	PDO	RFANTH	RFSOLSTAT
1850	-0,374	0	-0,006280513	-0,153959385	0	-0,0972				-0,160239898	0
1851	-0,221	-0,00097612	-0,006940124	-0,154505598	-0,007	-0,067725				-0,162421842	-0,007
1852	-0,224	-0,001393631	-0,005699415	-0,15550631	-0,027	-0,038925				-0,162599356	-0,027
1853	-0,269	-0,000494756	-0,00428019	-0,156480738	-0,046	-0,01665				-0,161255683	-0,046
1854	-0,251	-0,000915213	-0,005522387	-0,159631526	-0,062	-0,00765				-0,166069127	-0,062
1855	-0,273	0,003736845	-0,006740752	-0,16026946	-0,068	-0,0549				-0,163273367	-0,068
1856	-0,353	0,01026074	-0,00689029	-0,161888362	-0,063	-1,043775				-0,158517912	-0,063
1857	-0,46	0,015462563	-0,006626884	-0,162350202	-0,044	-1,6245				-0,153514523	-0,044
1858	-0,466	0,021975417	-0,005780187	-0,162777903	-0,015	-0,783				-0,146582672	-0,015
1859	-0,287	0,027168429	-0,005465652	-0,164730938	0,015	-0,30195				-0,143028161	0,015
1860	-0,349	0,033670284	-0,006488817	-0,167209386	0,023	-0,123975				-0,140027919	0,023
1861	-0,413	0,038854519	-0,007771668	-0,168337999	0,005	-0,092025				-0,137255147	0,005
1862	-0,526	0,041614591	-0,009377441	-0,168995275	-0,019	-0,368775				-0,136758125	-0,019
1863	-0,281	0,044927598	-0,010940439	-0,171479612	-0,038	-0,27045				-0,137492453	-0,038
1864	-0,494	0,047685191	-0,011294395	-0,173982324	-0,054	-0,1251				-0,137591528	-0,054
1865	-0,274	0,052858462	-0,011756652	-0,175854968	-0,069	-0,054				-0,134753158	-0,069
1866	-0,249	0,056165192	-0,012409629	-0,176860869	-0,079	-0,022275	0,201	-0,16	0,355	-0,133105306	-0,079
1867	-0,322	0,058918261	-0,013201884	-0,180512657	-0,086	-0,0099	0,139	-0,03	0,203	-0,13479628	-0,086
1868	-0,234	0,06222149	-0,01343963	-0,18234866	-0,063	-0,005625	0,152	-0,88	0,041	-0,133566801	-0,063
1869	-0,267	0,064972102	-0,013040149	-0,185558546	-0,029	-0,0162	0,093	0,55	-0,084	-0,133626593	-0,029
1870	-0,278	0,070131743	-0,012598665	-0,18714263	0,004	-0,016425	0,017	-0,15	-0,352	-0,129609552	0,004
1871	-0,334	0,074948943	-0,01396524	-0,190344563	-0,002	-0,015975	0,031	-0,15	-0,281	-0,12936086	-0,002
1872	-0,23	0,077694483	-0,013571333	-0,196310913	-0,008	-0,03555	0,093	1,91	-0,568	-0,132187762	-0,008
1873	-0,304	0,082844123	-0,011636958	-0,200331545	-0,037	-0,081225	0,047	0,08	-0,619	-0,12912438	-0,037
1874	-0,375	0,087441968	-0,010321209	-0,198255892	-0,059	-0,052875	-0,022	0,59	-0,338	-0,121135133	-0,059
1875	-0,397	0,092584296	-0,008519207	-0,201945988	-0,085	-0,034875	0,033	0,27	-0,603	-0,117880899	-0,085
1876	-0,382	0,095531652	-0,007678257	-0,203794344	-0,094	-0,1674	-0,023	0,44	-1,171	-0,115940949	-0,094
1877	-0,076	0,100121258	-0,005968311	-0,206366448	-0,092	-0,1422	0,241	-1,8	-0,065	-0,112213501	-0,092
1878	0,036	0,11265084	-0,00353805	-0,206507527	-0,092	-0,085275	0,451	0,17	0,512	-0,097394737	-0,092
1879	-0,232	0,124610937	-0,003031549	-0,212597216	-0,084	-0,054	0,123	1,27	-0,099	-0,091017827	-0,084
1880	-0,229	0,138932591	-0,005613529	-0,221431107	-0,062	-0,030825	0,061	0,79	-1,024	-0,088112045	-0,062
1881	-0,207	0,1513862	-0,004855182	-0,224690967	-0,041	-0,018	0,041	-0,59	0,305	-0,078159949	-0,041
1882	-0,214	0,163815737	-0,004080631	-0,230587953	-0,036	-0,015525	-0,027	-0,6	-1,203	-0,070852846	-0,036
1883	-0,298	0,174392554	-0,00589995	-0,237058341	-0,03	-1,276875	-0,033	-0,25	-0,961	-0,068565737	-0,03
1884	-0,411	0,183126107	-0,004366421	-0,239607931	-0,05	-3,858525	-0,077	-0,38	0,235	-0,060848245	-0,05
1885	-0,39	0,191306843	-0,00148535	-0,240993757	-0,073	-1,714275	-0,026	-0,68	1,341	-0,051172264	-0,073
1886	-0,368	0,202380461	0,000840958	-0,242751447	-0,105	-0,9837	0,121	0,7	-0,085	-0,039530028	-0,105
1887	-0,422	0,207439354	0,001886315	-0,247932292	-0,118	-1,001925	0,121	0,47	-0,675	-0,038606623	-0,118
1888	-0,312	0,210338114	0,000424014	-0,260895034	-0,127	-0,590175	0,193	-1,27	0,285	-0,050132907	-0,127
1889	-0,173	0,213572456	0,000989324	-0,259430538	-0,13	-0,7704	0,195	0,16	0,163	-0,044868758	-0,13
1890	-0,417	0,218622391	0,002362167	-0,272829406	-0,121	-1,056375	-0,147	0,56	0,03	-0,051844847	-0,121
1891	-0,335	0,22366851	0,001533785	-0,280150338	-0,08	-0,809325	0,034	-0,28	0,423	-0,054948043	-0,08
1892	-0,456	0,229247434	0,00267152	-0,284372683	-0,063	-0,584775	-0,094	0,52	-0,787	-0,052453728	-0,063
1893	-0,473	0,22884186	0,005147549	-0,284454104	-0,038	-0,254925	-0,007	0,99	-1,017	-0,050464695	-0,038
1894	-0,405	0,231727959	0,008237449	-0,28685377	-0,02	-0,0954	-0,246	0,21	-1,698	-0,046888362	-0,02
1895	-0,39	0,233134884	0,010880709	-0,297348956	-0,036	-0,0369	-0,096	-0,25	-0,183	-0,053333363	-0,036
1896	-0,184	0,236551604	0,013917385	-0,301852045	-0,065	-0,49365	0,105	-1,55	0,569	-0,051383056	-0,065
1897	-0,208	0,237955322	0,018705209	-0,309267882	-0,091	-0,455175	0,108	-0,68	0,344	-0,052607351	-0,091
1898	-0,413	0,239357727	0,023753855	-0,319698465	-0,099	-0,32715	0,073	0,57	-0,103	-0,056586883	-0,099
1899	-0,289	0,248205178	0,027303868	-0,335296372	-0,108	-0,123075	0,127	0,16	-0,463	-0,059787326	-0,108
1900	-0,198	0,256846378	0,029885359	-0,344432695	-0,112	-0,047475	0,096	-0,65	1,036	-0,057700958	-0,112
1901	-0,261	0,26834277	0,036509385	-0,354268971	-0,118	-0,020025	0,086	0,07	0,037	-0,049416816	-0,118
1902	-0,404	0,278015473	0,041577912	-0,360592498	-0,109	-0,5454	-0,103	0,1	0,978	-0,040999112	-0,109
1903	-0,482	0,287673509	0,042414156	-0,379900207	-0,07	-1,93095	-0,195	0,47	0,438	-0,049812542	-0,07
1904	-0,519	0,299641335	0,045560575	-0,381961874	-0,025	-0,858375	-0,348	0,39	0,661	-0,036759964	-0,025
1905	-0,377	0,311062202	0,049031632	-0,397308308	-0,053	-0,339525	-0,203	-1,79	1,421	-0,037214474	-0,053
1906	-0,284	0,321193675	0,052840113	-0,410498343	-0,022	-0,19575	-0,074	0,21	0,884	-0,036464555	-0,022
1907	-0,466	0,33078646	0,055637372	-0,437005891	-0,045	-0,2484	-0,227	-0,2	0,535	-0,050582059	-0,045
1908	-0,511	0,343009608	0,060348178	-0,424105628	-0,041	-0,277425	-0,13	0,28	0,582	-0,020747842	-0,041
1909	-0,523	0,353089143	0,06532668	-0,438413156	-0,063	-0,108225	-0,137	0,29	-0,354	-0,019997333	-0,063
1910	-0,491	0,363488138	0,066112873	-0,451841936	-0,083	-0,084375	-0,248	1,26	-0,349	-0,022240925	-0,083
1911	-0,543	0,375314138	0,065364006	-0,459539785	-0,093	-0,045225	-0,213	-0,64	-0,295	-0,018861641	-0,093
1912	-0,437	0,385677148	0,063760591	-0,477124434	-0,095	-0,5211	-0,228	-0,98	0,083	-0,027686695	-0,095
1913	-0,425	0,394248074	0,061330575	-0,499293639	-0,087	-0,65025	-0,387	-0,68	0,271	-0,04371499	-0,087
1914	-0,245	0,399433328	0,059882004	-0,476870084	-0,074	-0,26775	-0,289	-0,93	-0,147	-0,017554752	-0,074
1915	-0,147	0,406207933	0,058752162	-0,482478565	-0,027	-0,106425	0,096	0,16	0,318	-0,01751847	-0,04763303
1916	-0,382	0,417025908	0,056010859	-0,504151007	0,005	-0,074025	-0,075	0,64	-1,369	-0,03111424	-0,01926546
1917	-0,463	0,427317467	0,054618241	-0,515941858	0,025	-0,060075	-0,278	2,14	-1,124	-0,03400615	-0,00681081
1918	-0,331	0,438433681	0,053686022	-0,512984121	0,021	-0,05265	-0,259	0,05	0,203	-0,020864417	-0,030805
1919	-0,272	0,445665012	0,055370484	-0,462282429	-0,014	-0,054225	-0,186	-1,09	-0,332	0,038753068	-0,05753321
1920	-0,241	0,456412089	0,058018197	-0,491278669	-0,036	-0,252675	-0,333	0,09	-1,207	0,023151617	-0,07290454
1921	-0,187	0,465212907	0,066424556	-0,446639206	-0,046	-0,206775	-0,218	0,66	-1,021	0,084998257	-0,09922976
1922	-0,301	0,475925858	0,072646748	-0,465463368	-0,057	-0,078075	-0,318	0,33	-0,596	0,083109239	-0,11076099
1923	-0,272	0,486620016	0,066093192	-0,525395742	-0,046	-0,03015	-0,321	-0,36	-0,07	0,027317466	-0,10257205
1924	-0,292	0,497295491	0,063272571	-0,523332517	-0,039	-0,092025	-0,15	0,34	-0,783	0,037235545	-0,09948294
1925	-0,214	0,508447143	0,065664731	-0,533393617	-0,007	-0,077175	-0,156	-0,24	-0,255	0,040718257	-0,06199333
1926	-0,105	0,520833867	0,067164833	-0,540015123	0,009	-0,063225	0,088	-0,49	1,628	0,047983577	-0,03175666
1927	-0,208	0,531784323	0,069791647	-0,557067927	0,039	-0,040725	0,113	0,27	-0,103	0,044508043	-0,02098404
1928	-0,206	0,542048206	0,071380148	-0,55569895	0,021	-0,14175	-0,002	0,42	-0,088	0,057729404	-0,00625254
1929	-0,348	0,556112322	0,070422724	-0,577628605	0,017	-0,265275	-0,109	0,46	0,4	0,048906441	-0,01739218
1930	-0,134	0,564442719	0,070770353	-0,547173604	0,021	-0,167625	0,015	0,03	0,02	0,088039468	-0,06053392

1931	-0,083	0,576389369	0,075219255	-0,504134681	0,01	-0,1269	0,183	0,39	0,746	0,147473943	-0,07657757
1932	-0,134	0,584692687	0,080070364	-0,46154691	-0,001	-0,222525	0,232	-0,68	-0,601	0,203216141	-0,11597844
1933	-0,267	0,598666752	0,081829642	-0,480538096	-0,016	-0,180225	0,197	0,09	-1,474	0,199958298	-0,10377671
1934	-0,127	0,60867146	0,080991833	-0,512626663	0,003	-0,1026	-0,002	-0,01	0,776	0,17703663	-0,11533023
1935	-0,172	0,618661159	0,080356327	-0,538778787	0,035	-0,1143	0,023	0,14	0,499	0,160238699	-0,07967578
1936	-0,14	0,628635916	0,081022315	-0,560945216	0,092	-0,087975	0,154	0,03	1,648	0,148713015	-0,05695532
1937	-0,022	0,640318543	0,082293885	-0,586432345	0,078	-0,07605	0,299	0,08	-0,182	0,136180083	-0,01798878
1938	-0,003	0,650206986	0,089884902	-0,55361719	0,069	-0,132525	0,245	0,87	-0,109	0,186474698	-0,01210266
1939	-0,047	0,656698003	0,096115825	-0,573278146	0,069	-0,1107	0,113	0,02	-0,356	0,179535682	-0,01414587
1940	0,018	0,661605174	0,094048785	-0,603525567	0,067	-0,086175	-0,025	-1,52	1,723	0,152128393	-0,02031331
1941	0,02	0,659959842	0,092916645	-0,623590914	0,065	-0,052425	0,178	-1,45	2,427	0,129285573	-0,06300316
1942	-0,024	0,658105439	0,095249742	-0,644946096	0,061	-0,1134	0,186	0,02	0,422	0,108409085	-0,08882759
1943	0	0,65768683	0,097791679	-0,655648463	0,046	-0,118125	0,036	0,36	-0,82	0,099830047	-0,09431893
1944	0,149	0,655811305	0,098226406	-0,652871959	0,057	-0,06345	0,352	-0,28	-0,817	0,101165751	-0,1265606
1945	0,027	0,661626546	0,095557532	-0,614189581	0,097	-0,05985	0,222	0,42	-0,448	0,142994496	-0,08842031
1946	-0,072	0,664634738	0,097800623	-0,600290801	0,107	-0,04905	0,021	-0,79	-0,417	0,16214456	-0,08695959
1947	-0,038	0,670900159	0,101956298	-0,641819198	0,141	-0,06165	-0,082	0,16	0,583	0,131037259	-0,04376538
1948	-0,037	0,67662697	0,099978685	-0,650241626	0,164	-0,046125	0,015	-0,23	-0,562	0,126364029	-0,0211973
1949	-0,072	0,688250337	0,1005346	-0,629957646	0,149	-0,0882	0,094	-0,21	-1,531	0,158827292	-0,00627244
1950	-0,172	0,699304614	0,102913766	-0,66930899	0,117	-0,0774	-0,006	1,51	-2,083	0,132909391	-0,01961315
1951	-0,051	0,712783473	0,119495107	-0,697788265	0,082	-0,053325	0,206	-0,69	-1,038	0,134490314	-0,03153129
1952	0,031	0,724732237	0,138587275	-0,71837546	0,083	-0,099225	0,297	-0,22	-1,396	0,144944052	-0,05429477
1953	0,097	0,738145705	0,140099149	-0,735053463	0,061	-0,091575	0,266	-0,77	-0,551	0,14319139	-0,10518662
1954	-0,129	0,753909869	0,141379365	-0,756149911	0,065	-0,097425	0,045	0,23	-0,371	0,139139324	-0,12175511
1955	-0,19	0,76864185	0,148537329	-0,810647669	0,082	-0,0486	0,195	0,9	-2,035	0,10653151	-0,09885812
1956	-0,266	0,785365315	0,156881598	-0,859463225	0,157	-0,0306	-0,017	1,02	-1,747	0,082783687	-0,09471641
1957	-0,004	0,800663386	0,162195123	-0,896854702	0,209	-0,01395	0,043	-0,45	0,347	0,066003807	-0,08132834
1958	0,046	0,816645925	0,168476026	-0,92218268	0,205	-0,00675	0,215	-0,5	0,881	0,062939271	-0,04705626
1959	0,017	0,837987311	0,17232438	-0,966152564	0,169	-0,0054	0,054	-0,1	0,322	0,044159126	-0,03178992
1960	-0,049	0,861181847	0,174174045	-1,019591274	0,157	-0,12465	0,237	0,29	0,046	0,015764618	-0,01760758
1961	0,04	0,880221948	0,178718268	-1,044643109	0,103	-0,290925	0,102	-0,01	-0,654	0,014297107	-0,02471357
1962	0,016	0,900404071	0,180415509	-1,077647666	0,072	-0,35775	0,074	0,39	-1,282	0,003171914	-0,04388815
1963	0,049	0,920229252	0,181709944	-1,122111664	0,064	-1,2411	0,005	-0,32	-0,372	-0,020172468	-0,0779682
1964	-0,223	0,939133014	0,185578621	-1,166615475	0,068	-1,935675	-0,094	0,54	-0,737	-0,04190384	-0,09930591
1965	-0,14	0,959178832	0,191438394	-1,202952705	0,077	-1,16595	-0,157	-0,95	-0,115	-0,052335479	-0,1222086
1966	-0,069	0,992323304	0,19842406	-1,238686851	0,109	-0,6273	0,006	-0,54	-0,491	-0,047939486	-0,10042686
1967	-0,074	1,015458345	0,200693254	-1,251632075	0,14	-0,3915	-0,097	0,25	-0,758	-0,035480476	-0,10754799
1968	-0,112	1,038818901	0,201581727	-1,310163398	0,151	-0,740025	-0,165	0,2	-0,159	-0,069762771	-0,05656062
1969	0,031	1,070649962	0,208498157	-1,363075161	0,166	-0,9288	0,011	-0,66	-0,357	-0,083927042	-0,04297371
1970	-0,027	1,10475302	0,221660749	-1,410992472	0,164	-0,4473	-0,102	0,29	-0,359	-0,084578703	-0,03140636
1971	-0,187	1,131312497	0,228750575	-1,4089187	0,117	-0,17505	-0,311	1,07	-1,378	-0,048855628	-0,01541193
1972	-0,067	1,161729297	0,223103944	-1,426579377	0,123	-0,105975	-0,353	-0,83	-1,189	-0,041746136	-0,01330933
1973	0,062	1,20758088	0,220826421	-1,462172379	0,081	-0,210375	-0,214	0,64	-1,206	-0,033765078	-0,03463157
1974	-0,213	1,233519159	0,220978305	-1,442512066	0,069	-0,343125	-0,421	0,96	-0,294	0,011985398	-0,07110029
1975	-0,147	1,264506043	0,217084922	-1,420887644	0,043	-0,813375	-0,298	1,33	-1,389	0,060703321	-0,09289115
1976	-0,24	1,285618021	0,220927438	-1,447358797	0,064	-0,367875	-0,363	0,06	-0,095	0,059186662	-0,10098143
1977	0,046	1,327954805	0,227878831	-1,45755076	0,096	-0,137475	-0,188	-1,14	0,071	0,098282876	-0,12302647
1978	-0,063	1,366650802	0,231222895	-1,451750832	0,16	-0,203625	-0,179	-0,29	0,105	0,146122865	-0,09788582
1979	0,058	1,405415955	0,240561404	-1,462380829	0,212	-0,2475	-0,109	-0,26	0,119	0,183596529	-0,07763544
1980	0,093	1,445857586	0,23770971	-1,441416039	0,214	-0,1269	-0,017	-0,43	0,332	0,242151257	-0,04483984
1981	0,14	1,481919344	0,23306174	-1,415985056	0,219	-0,134775	-0,075	0,07	0,881	0,298996028	-0,00969692
1982	0,011	1,514529923	0,249065235	-1,398870906	0,155	-1,418175	-0,212	-1,45	-0,173	0,364724252	-0,00476562
1983	0,193	1,541905861	0,262543019	-1,388528971	0,145	-2,031525	-0,069	-0,95	1,262	0,415919909	-0,0119908
1984	-0,013	1,580143571	0,271446225	-1,394661112	0,088	-0,814275	-0,206	-0,14	0,683	0,456928684	-0,04510844
1985	-0,03	1,61201841	0,282654251	-1,403162369	0,065	-0,3411	-0,265	-0,02	0,09	0,491510292	-0,0892912
1986	0,046	1,645783012	0,292276565	-1,407791093	0,065	-0,3663	-0,272	-0,32	1,03	0,530268484	-0,08913905
1987	0,191	1,679371738	0,297864928	-1,421333433	0,087	-0,279	0,071	-1,48	1,213	0,555903234	-0,1044477
1988	0,199	1,727614372	0,302146771	-1,425069766	0,128	-0,205425	-0,002	0,74	-0,022	0,604691377	-0,11449083
1989	0,118	1,764780608	0,307138862	-1,433794003	0,208	-0,165825	-0,079	0,62	-0,432	0,638125467	-0,08709761
1990	0,296	1,789194711	0,310875246	-1,42	0,196	-0,1656	-0,034	-0,33	-0,722	0,680069957	-0,04584147
1991	0,254	1,818018618	0,320250855	-1,400996984	0,178	-1,45575	-0,129	-1,01	-0,774	0,737272489	-0,02592497
1992	0,103	1,837891616	0,316998909	-1,370512619	0,16	-3,270825	-0,216	-1,23	0,806	0,784377906	-0,00441801
1993	0,145	1,85223471	0,302561809	-1,354311622	0,122	-1,323	-0,207	-1,11	1,068	0,800484897	-0,01389205
1994	0,206	1,877431425	0,294434886	-1,337991932	0,088	-0,53865	-0,172	-1,35	-0,485	0,833874379	-0,0269047
1995	0,321	1,914544406	0,293025013	-1,320665137	0,074	-0,2601	0,141	-0,33	0,3	0,886904281	-0,05017639
1996	0,18	1,946177527	0,29708327	-1,3167814	0,061	-0,1755	-0,054	0,56	0,573	0,926479396	-0,08785955
1997	0,389	1,963474132	0,28980048	-1,308417237	0,08	-0,14085	0,057	-1,29	1,176	0,944857375	-0,09697299
1998	0,536	2,011001988	0,27408101	-1,29386044	0,132	-0,0756	0,378	-0,23	-0,431	0,991222558	-0,11340304
1999	0,306	2,047719178	0,264176124	-1,262894038	0,173	-0,05715	0,124	0,79	-1,874	1,049001264	-0,10182793
2000	0,293	2,067603772	0,25935132	-1,260281183	0,213	-0,0567	0,034	0,72	-1,123	1,06667391	-0,08990334
2001	0,439	2,091246864	0,260600715	-1,24720891	0,19870059	-0,056925	0,125	-0,08	-1,209	1,104638668	-0,06582002
2002	0,497	2,122853338	0,267001206	-1,242265453	0,20865272	-0,056475	0,071	-0,72	-0,515	1,147589091	-0,02929697
2003	0,508	2,162905998	0,272495477	-1,264297356	0,12634931	-0,0729	0,239	-0,43	0,206	1,171104119	-0,01678344
2004	0,448	2,190358436	0,277499695	-1,292141239	0,08875129	-0,065475	0,213	-0,63	-0,307	1,175716892	-0,00475987
2005	0,544	2,220183658	0,281093644	-1,30294793	0,05482068	-0,0927	0,3	-0,46	-0,265	1,198329373	-0,02131667
2006	0,505	2,253150576	0,281873419	-1,302047517	0,04145274	-0,104175	0,275	-0,24	-0,329	1,232976478	-0,05156956
2007	0,492	2,282359347	0,281247912	-1,293334498	0,02160381	-0,11745	0,154	0,03	-0,715	1,270272761	-0,09596315
2008	0,394	2,316320707	0,280626603	-1,275121104	0,01268869	-0,102375	0,146	1	-1,642	1,321826206	-0,12053198
2009	0,506	2,341321577	0,280007273	-1,239945175	0,00932964	-0,126225	0,049	-0,13	-0,972	1,381383676	-0,11173045
2010	0,556	2,376924117	0,278824757	-1,231569268	0,04509787	-0,0963	0,359	0,95	-1,045	1,424179606	-0,10931546
2011	0,421	2,40815158	0,276515877	-1,21568537	0,08282235	-0,132975	0,111	1,38	-1,806	1,468982087	-0,06737349

Dataset of sensitivity runs

Year	HADCRUT4	RFANTHCMIP5	RFWARMCMIP5	RFSOXCMIP5	RFANTHANSEN	RFWARMHANSEN	RFSOXHANSEN
1850	-0,374	0	0	0	0	0	0
1851	-0,221	0,007247197	0,00660299	0,000644207	0	0	0
1852	-0,224	0,011795687	0,01115148	0,000644207	0	0	0
1853	-0,269	0,015880087	0,01523588	0,000644207	0	0	0
1854	-0,251	0,021814487	0,02117028	0,000644207	0	0	0
1855	-0,273	0,026075787	0,02543158	0,000644207	0	0	0
1856	-0,353	0,031432687	0,03078848	0,000644207	0	0	0
1857	-0,46	0,036257487	0,03561328	0,000644207	0	0	0
1858	-0,466	0,042020887	0,04137668	0,000644207	0	0	0
1859	-0,287	0,048232487	0,04758828	0,000644207	0	0	0
1860	-0,349	0,052598387	0,05195418	0,000644207	0	0	0
1861	-0,413	0,058548187	0,05790398	0,000644207	0	0	0
1862	-0,526	0,062636187	0,06199198	0,000644207	0	0	0
1863	-0,281	0,067203387	0,06655918	0,000644207	0	0	0
1864	-0,494	0,072679887	0,07203568	0,000644207	0	0	0
1865	-0,274	0,077364487	0,07672028	0,000644207	0	0	0
1866	-0,249	0,081770887	0,08112668	0,000644207	0	0	0
1867	-0,322	0,085595887	0,08495168	0,000644207	0	0	0
1868	-0,234	0,090158487	0,08951428	0,000644207	0	0	0
1869	-0,267	0,095668087	0,09502388	0,000644207	0	0	0
1870	-0,278	0,100745687	0,10010148	0,000644207	0	0	0
1871	-0,334	0,106765387	0,10612118	0,000644207	0	0	0
1872	-0,23	0,112705387	0,11206118	0,000644207	0	0	0
1873	-0,304	0,119472387	0,11882818	0,000644207	0	0	0
1874	-0,375	0,127395387	0,12675118	0,000644207	0	0	0
1875	-0,397	0,136120387	0,13547618	0,000644207	0	0	0
1876	-0,382	0,145333167	0,14468896	0,000644207	0	0	0
1877	-0,076	0,155865557	0,15522135	0,000644207	0	0	0
1878	0,036	0,167212537	0,16656833	0,000644207	0	0	0
1879	-0,232	0,178320067	0,17767586	0,000644207	0	0	0
1880	-0,229	0,191657447	0,19101324	0,000644207	0	0	0
1881	-0,207	0,205351507	0,2047073	0,000644207	0,006	0,017	-0,011
1882	-0,214	0,218781007	0,2181368	0,000644207	0,012	0,034	-0,022
1883	-0,298	0,230839557	0,23019535	0,000644207	0,017	0,05	-0,033
1884	-0,411	0,243863497	0,24321929	0,000644207	0,019	0,063	-0,044
1885	-0,39	0,254639967	0,25399576	0,000644207	0,021	0,076	-0,055
1886	-0,368	0,264114637	0,26347043	0,000644207	0,025	0,091	-0,066
1887	-0,422	0,273146917	0,27250271	0,000644207	0,024	0,1	-0,076
1888	-0,312	0,280255787	0,27961158	0,000644207	0,02	0,107	-0,087
1889	-0,173	0,287376357	0,28673215	0,000644207	0,015	0,114	-0,099
1890	-0,417	0,294290596	0,29365106	0,000639536	0,014	0,123	-0,109
1891	-0,335	0,29648054	0,29933554	-0,002855	0,012	0,132	-0,12
1892	-0,456	0,29758027	0,30671103	-0,00913076	0,009	0,141	-0,132
1893	-0,473	0,29773701	0,31211664	-0,01437963	0,003	0,145	-0,142
1894	-0,405	0,29992503	0,3170119	-0,01708687	-0,001	0,152	-0,153
1895	-0,39	0,29932034	0,3220897	-0,02276936	-0,007	0,157	-0,164
1896	-0,184	0,30167273	0,3275001	-0,02582737	-0,011	0,164	-0,175
1897	-0,208	0,30320702	0,3328641	-0,02965708	-0,017	0,169	-0,186
1898	-0,413	0,30592067	0,340105	-0,03418433	-0,023	0,174	-0,197
1899	-0,289	0,31207837	0,3495209	-0,03744253	-0,02	0,188	-0,208
1900	-0,198	0,31844533	0,3591227	-0,04067737	-0,018	0,201	-0,219
1901	-0,261	0,3251553	0,3700978	-0,0449425	-0,013	0,216	-0,229
1902	-0,404	0,3335775	0,3826493	-0,0490718	-0,01	0,229	-0,239
1903	-0,482	0,3408752	0,3942656	-0,0533904	-0,006	0,243	-0,249
1904	-0,519	0,3505828	0,4077738	-0,057191	0	0,259	-0,259
1905	-0,377	0,3592629	0,4213288	-0,0620659	0,005	0,274	-0,269
1906	-0,284	0,3717691	0,4368834	-0,0651143	0,009	0,288	-0,279
1907	-0,466	0,3799316	0,4504954	-0,0705638	0,011	0,301	-0,29
1908	-0,511	0,3920956	0,4646723	-0,0725767	0,017	0,317	-0,3
1909	-0,523	0,3999937	0,4787183	-0,0787246	0,021	0,331	-0,31
1910	-0,491	0,4096997	0,492886	-0,0831863	0,026	0,346	-0,32
1911	-0,543	0,4233188	0,5059278	-0,082609	0,031	0,361	-0,33
1912	-0,437	0,439408	0,5185576	-0,0791496	0,035	0,375	-0,34
1913	-0,425	0,4542059	0,5326287	-0,0784228	0,037	0,387	-0,35
1914	-0,245	0,4685044	0,5455269	-0,0770225	0,035	0,395	-0,36
1915	-0,147	0,4802106	0,5579125	-0,0777019	0,035	0,406	-0,371
1916	-0,382	0,4951513	0,5710789	-0,0759276	0,039	0,42	-0,381
1917	-0,463	0,5092378	0,5851528	-0,075915	0,043	0,434	-0,391
1918	-0,331	0,5239102	0,598776	-0,0748658	0,048	0,449	-0,401
1919	-0,272	0,5369487	0,6121328	-0,0751841	0,049	0,46	-0,411
1920	-0,241	0,5521736	0,6254585	-0,0732849	0,053	0,475	-0,422
1921	-0,187	0,5593754	0,640022	-0,0806466	0,055	0,487	-0,432
1922	-0,301	0,5696386	0,6550556	-0,085417	0,06	0,502	-0,442
1923	-0,272	0,5796128	0,6686489	-0,0890361	0,065	0,517	-0,452
1924	-0,292	0,5930275	0,6837707	-0,0907432	0,07	0,532	-0,462
1925	-0,214	0,6030189	0,7004647	-0,0974458	0,074	0,546	-0,472
1926	-0,105	0,6142638	0,7162835	-0,1020197	0,081	0,562	-0,481
1927	-0,208	0,6224751	0,732189	-0,1097139	0,086	0,577	-0,491
1928	-0,206	0,6340874	0,7491961	-0,1151087	0,09	0,591	-0,501
1929	-0,348	0,6444652	0,766149	-0,1216838	0,096	0,607	-0,511
1930	-0,134	0,6535576	0,7811643	-0,1276067	0,1	0,62	-0,52

1931	-0,083	0,6651429	0,7978251	-0,1326822	0,106	0,635	-0,529
1932	-0,134	0,6779398	0,8141178	-0,136178	0,109	0,648	-0,539
1933	-0,267	0,6902062	0,829371	-0,1391648	0,116	0,665	-0,549
1934	-0,127	0,702913	0,8439186	-0,1410056	0,119	0,678	-0,559
1935	-0,172	0,7160091	0,8582087	-0,1421996	0,123	0,691	-0,568
1936	-0,14	0,7248916	0,872135	-0,1472434	0,128	0,705	-0,577
1937	-0,022	0,7328986	0,8829384	-0,1500398	0,133	0,72	-0,587
1938	-0,003	0,7389094	0,8925516	-0,1536422	0,137	0,734	-0,597
1939	-0,047	0,7454794	0,900972	-0,1554926	0,138	0,745	-0,607
1940	0,018	0,7468345	0,9086638	-0,1618293	0,134	0,75	-0,616
1941	0,02	0,7499876	0,9156286	-0,165641	0,124	0,75	-0,626
1942	-0,024	0,7465792	0,9203051	-0,1737259	0,116	0,751	-0,635
1943	0	0,74270573	0,9246475	-0,18194177	0,108	0,753	-0,645
1944	0,149	0,7388164	0,9293959	-0,1905795	0,099	0,754	-0,655
1945	0,027	0,73740832	0,936577	-0,19916868	0,097	0,761	-0,664
1946	-0,072	0,73654751	0,9437328	-0,20718529	0,094	0,768	-0,674
1947	-0,038	0,74072139	0,9540803	-0,21335891	0,096	0,779	-0,683
1948	-0,037	0,74310569	0,9644172	-0,22131151	0,095	0,788	-0,693
1949	-0,072	0,74804026	0,9775409	-0,22950064	0,1	0,803	-0,703
1950	-0,172	0,75449799	0,9923465	-0,23784851	0,107	0,819	-0,712
1951	-0,051	0,7592571	1,0089371	-0,24968	0,103	0,841	-0,738
1952	0,031	0,7649993	1,0273956	-0,2623963	0,099	0,862	-0,763
1953	0,097	0,7740853	1,0480619	-0,2739766	0,094	0,884	-0,79
1954	-0,129	0,7824154	1,0697794	-0,287364	0,093	0,909	-0,816
1955	-0,19	0,7924332	1,094053	-0,3016198	0,093	0,934	-0,841
1956	-0,266	0,8046171	1,119343	-0,3147259	0,091	0,958	-0,867
1957	-0,004	0,816751	1,1453344	-0,3285834	0,093	0,986	-0,893
1958	0,046	0,8305234	1,171722	-0,3411986	0,092	1,011	-0,919
1959	0,017	0,8514493	1,2005387	-0,3490894	0,099	1,044	-0,945
1960	-0,049	0,8720615	1,2321618	-0,3601003	0,108	1,079	-0,971
1961	0,04	0,8873409	1,2641439	-0,376803	0,113	1,109	-0,996
1962	0,016	0,9017542	1,2950439	-0,3932897	0,12	1,14	-1,02
1963	0,049	0,9148941	1,3249293	-0,4100352	0,129	1,173	-1,044
1964	-0,223	0,9290152	1,3544312	-0,425416	0,135	1,204	-1,069
1965	-0,14	0,9477325	1,3883752	-0,4406427	0,144	1,237	-1,093
1966	-0,069	0,9727761	1,4282447	-0,4554686	0,17	1,288	-1,118
1967	-0,074	0,9974402	1,4692678	-0,4718276	0,184	1,326	-1,142
1968	-0,112	1,0261323	1,5123151	-0,4861828	0,199	1,366	-1,167
1969	0,031	1,059195	1,5603449	-0,5011499	0,225	1,416	-1,191
1970	-0,027	1,0900263	1,6070148	-0,5169885	0,253	1,469	-1,216
1971	-0,187	1,1161909	1,6510176	-0,5348267	0,271	1,514	-1,243
1972	-0,067	1,1473136	1,7008705	-0,5535569	0,295	1,564	-1,269
1973	0,062	1,1870997	1,7572236	-0,5701239	0,336	1,631	-1,295
1974	-0,213	1,2201594	1,8062649	-0,5861055	0,357	1,678	-1,321
1975	-0,147	1,2554864	1,854175	-0,5986886	0,382	1,73	-1,348
1976	-0,24	1,2927096	1,9055486	-0,612839	0,395	1,769	-1,374
1977	0,046	1,3350053	1,9652853	-0,63028	0,434	1,835	-1,401
1978	-0,063	1,3803525	2,0252785	-0,644926	0,467	1,893	-1,426
1979	0,058	1,4293798	2,0875138	-0,658134	0,502	1,955	-1,453
1980	0,093	1,4782777	2,1518077	-0,67353	0,537	2,016	-1,479
1981	0,14	1,5155339	2,2083599	-0,692826	0,578	2,087	-1,509
1982	0,011	1,5499824	2,2617404	-0,711758	0,614	2,152	-1,538
1983	0,193	1,5845589	2,3185509	-0,733992	0,643	2,211	-1,568
1984	-0,013	1,6238774	2,3766324	-0,752755	0,685	2,283	-1,598
1985	-0,03	1,6615277	2,4315747	-0,770047	0,721	2,348	-1,627
1986	0,046	1,6975955	2,4849515	-0,787356	0,761	2,419	-1,658
1987	0,191	1,7369583	2,5431383	-0,80618	0,801	2,488	-1,687
1988	0,199	1,7836939	2,6067399	-0,823046	0,859	2,576	-1,717
1989	0,118	1,8277963	2,6665273	-0,838731	0,902	2,648	-1,746
1990	0,296	1,8636816	2,7175666	-0,853885	0,931	2,707	-1,776
1991	0,254	1,9020236	2,7594866	-0,857463	0,947	2,764	-1,817
1992	0,103	1,9301736	2,7941466	-0,863973	0,949	2,804	-1,855
1993	0,145	1,9568936	2,8243866	-0,867493	0,943	2,836	-1,893
1994	0,206	1,9888446	2,8590466	-0,870202	0,949	2,879	-1,93
1995	0,321	2,0250106	2,8991566	-0,874146	0,969	2,934	-1,965
1996	0,18	2,0634826	2,9391266	-0,875644	0,977	2,981	-2,004
1997	0,389	2,1021816	2,9803566	-0,878175	0,967	3,012	-2,045
1998	0,536	2,1470916	3,0274266	-0,880335	0,994	3,078	-2,084
1999	0,306	2,1890156	3,0718166	-0,882801	1,01	3,13	-2,12
2000	0,293	2,2198236	3,1069366	-0,887113	1,005	3,164	-2,159
2001	0,439	2,2536366	3,1404166	-0,88678	1,012	3,197	-2,185
2002	0,497	2,2937666	3,1805466	-0,88678	1,032	3,239	-2,207
2003	0,508	2,3389266	3,2257066	-0,88678	1,06	3,29	-2,23
2004	0,448	2,3785166	3,2652966	-0,88678	1,052	3,307	-2,255
2005	0,544	2,4161366	3,3029166	-0,88678	1,067	3,346	-2,279
2006	0,505	2,4549566	3,3417366	-0,88678	1,089	3,392	-2,303
2007	0,492	2,4940066	3,3807866	-0,88678	1,106	3,436	-2,33
2008	0,394	2,5340366	3,4208166	-0,88678	1,124	3,483	-2,359
2009	0,506	2,5767866	3,4635666	-0,88678	1,133	3,521	-2,388
2010	0,556	2,6179766	3,5047566	-0,88678	1,156	3,573	-2,417
2011	0,421	2,6599366	3,5467166	-0,88678	1,172	3,62	-2,448
